# Cell-therapy for Parkinson’s disease: a systematic review and meta-analysis

**DOI:** 10.1186/s12967-023-04484-x

**Published:** 2023-09-07

**Authors:** Fang Wang, Zhengwu Sun, Daoyong Peng, Shikha Gianchandani, Weidong Le, Johannes Boltze, Shen Li

**Affiliations:** 1 Department of Neurology, Central Hospital of Dalian University of Technology, Dalian, China; 2 Department of Clinical Pharmacy, Central Hospital of Dalian University of Technology, Dalian, China; 3 School of Life Sciences, University of Warwick, Gibbet Hill Road, Coventry, CV4 7AL UK; 4 Institute of Neurology, Sichuan Academy of Medical Sciences, Sichuan Provincial Hospital, Chengdu, China; 5 Department of Neurology and Psychiatry, Beijing Shijitan Hospital, Capital Medical University, No. 10 Tieyi Road, Beijing, 100038 China; 6 Beijing Institute of Brain Disorders, Capital Medical University, Beijing, China

**Keywords:** Parkinson's disease, Cell-therapy, Homogenous cell, Transplantation, Meta-analysis

## Abstract

**Background:**

Cell-based strategies focusing on replacement or protection of dopaminergic neurons have been considered as a potential approach to treat Parkinson’s disease (PD) for decades. However, despite promising preclinical results, clinical trials on cell-therapy for PD reported mixed outcomes and a thorough synthesis of these findings is lacking. We performed a systematic review and meta-analysis to evaluate cell-therapy for PD patients.

**Methods:**

We systematically identified all clinical trials investigating cell- or tissue-based therapies for PD published before July 2023. Out of those, studies reporting transplantation of homogenous cells (containing one cell type) were included in meta-analysis. The mean difference or standardized mean difference in quantitative neurological scale scores before and after cell-therapy was analyzed to evaluate treatment effects.

**Results:**

The systematic literature search revealed 106 articles. Eleven studies reporting data from 11 independent trials (210 patients) were eligible for meta-analysis. Disease severity and motor function evaluation indicated beneficial effects of homogenous cell-therapy in the ‘off’ state at 3-, 6-, 12-, or 24-month follow-ups, and for motor function even after 36 months. Most of the patients were levodopa responders (61.6–100% in different follow-ups). Cell-therapy was also effective in improving the daily living activities in the ‘off’ state of PD patients. Cells from diverse sources were used and multiple transplantation modes were applied. Autografts did not improve functional outcomes, while allografts exhibited beneficial effects. Encouragingly, both transplantation into basal ganglia and to areas outside the basal ganglia were effective to reduce disease severity. Some trials reported adverse events potentially related to the surgical procedure. One confirmed and four possible cases of graft-induced dyskinesia were reported in two trials included in this meta-analysis.

**Conclusions:**

This meta-analysis provides preliminary evidence for the beneficial effects of homogenous cell-therapy for PD, potentially to the levodopa responders. Allogeneic cells were superior to autologous cells, and the effective transplantation sites are not limited to the basal ganglia.

*PROSPERO registration number*: CRD42022369760

**Supplementary Information:**

The online version contains supplementary material available at 10.1186/s12967-023-04484-x.

## Introduction

Parkinson’s disease (PD) is the second most common neurodegenerative disease, and no curative therapy is currently available [1]. Thus, alternative solutions are urgently needed. PD has long been considered to be among the most promising target diseases for cell replacement therapy due to the specific loss of dopaminergic neurons in the substantia nigra [2], and cell-based therapies for PD has been explored clinically during the past decades. Initial studies mostly focused on transplantation of tissues such as embryonic mesencephalic tissue, adrenal medulla tissue, carotid body tissue, and sympathetic ganglion tissue. A meta-analysis on tissue transplantation demonstrated improved functional outcome [3]. However, tissue transplantation has several shortcomings including severe graft-induced dyskinesia (GID), substantial outcome heterogeneity, unsurmountable difficulties in quality control, immunogenicity, and ethical restrictions. Therefore, researchers gradually switched to transplantation of homogenous cells (defined as cell populations containing only one cell type that was extracted, isolated, expanded, and characterized). These comprise neural progenitor cells, fetal stem cells, bone marrow mesenchymal stem cells, retinal pigment epithelial cells, or induced pluripotent stem cells. With the advances in regenerative medicine, engineered cells are being tested as well. Lately, implantation of autologous, induced pluripotent stem cell-derived midbrain dopaminergic progenitor cells was reported [4], which may help to overcome ethical concerns if used properly. Although homogeneous cell transplantation is translationally promising, mixed results were reported from individual trials and no meta-analysis of those results has been conducted so far. A meta-analysis is therefore necessary to provide an overall assessment of the safety and efficacy of cell-therapy approaches in PD. In this study, we systematically reviewed all clinical trials on tissue or cell transplantation for PD and performed a meta-analysis for homogenous cells in treatment of PD.

## Methods

This systematic review and meta-analysis was conducted according to the Preferred Reporting Items for Systematic Reviews and Meta-Analysis (PRISMA) guidelines [5].

### Search strategy

We systematically identified all clinical trials investigating cell-therapies for PD indexed in PubMed, Embase, Web of Science, and Cochrane databases before July 2023. The search terms were: *(Parkinson disease OR Parkinson’s disease OR Parkinsonian disorders OR Parkinsonism OR Parkinsonisms OR Parkinson OR Parkinsons) AND (cell therapy OR cell therapeutics OR cell treatment OR cell treatments OR transplantation OR implantation),* filtering for clinical trials. Only reports in English language were included.

FW and ZWS (review authors) screened studies for initial inclusion based on titles and abstracts. Full text screening for eligibility was performed if an initial decision could not be made. In case FW and ZWS could not reach a consensus, SL was consulted, followed by discussion and joint consensus in all cases. We also screened related reviews, together with reference lists of included publications, to identify other relevant articles [2, 6–9].

### Inclusion and exclusion criteria for the systematic review

The inclusion criteria were: (1) recruited patients were diagnosed with idiopathic PD; (2) cell or tissue transplantation; (3) randomized controlled trials (RCTs), open-label studies, cohort studies, case reports, prospective studies, or retrospective studies.

Exclusion criteria were: (1) trials focusing on secondary PD or Parkinsonism-plus syndrome; (2) transplantation of more than one tissue type; (3) reviews and book chapters.

### Additional inclusion and exclusion criteria for the meta-analysis

The studies included in the systematic review were further screened for the meta-analysis with the following inclusion criteria: (1) transplantation with homogenous cell populations (containing only one type of cells); (2) using objective methods to evaluate treatment responses such as imaging, biochemical indicators or quantitative scales, including Unified Parkinson Disease Rating Scale (UPDRS), or its part II/III (UPDRSII/UPDRSIII), Hoehn and Yahr (H&Y) Staging Scale, Beck Depression Inventory (BDI), Beck Anxiety Inventory, Mini-mental State Examination (MMSE), Parkinson's Disease Quality of Life Questionnaire, or Schwab and England Scale; (3) quantitative data available before and after cell-therapy.

**Fig. 1:**
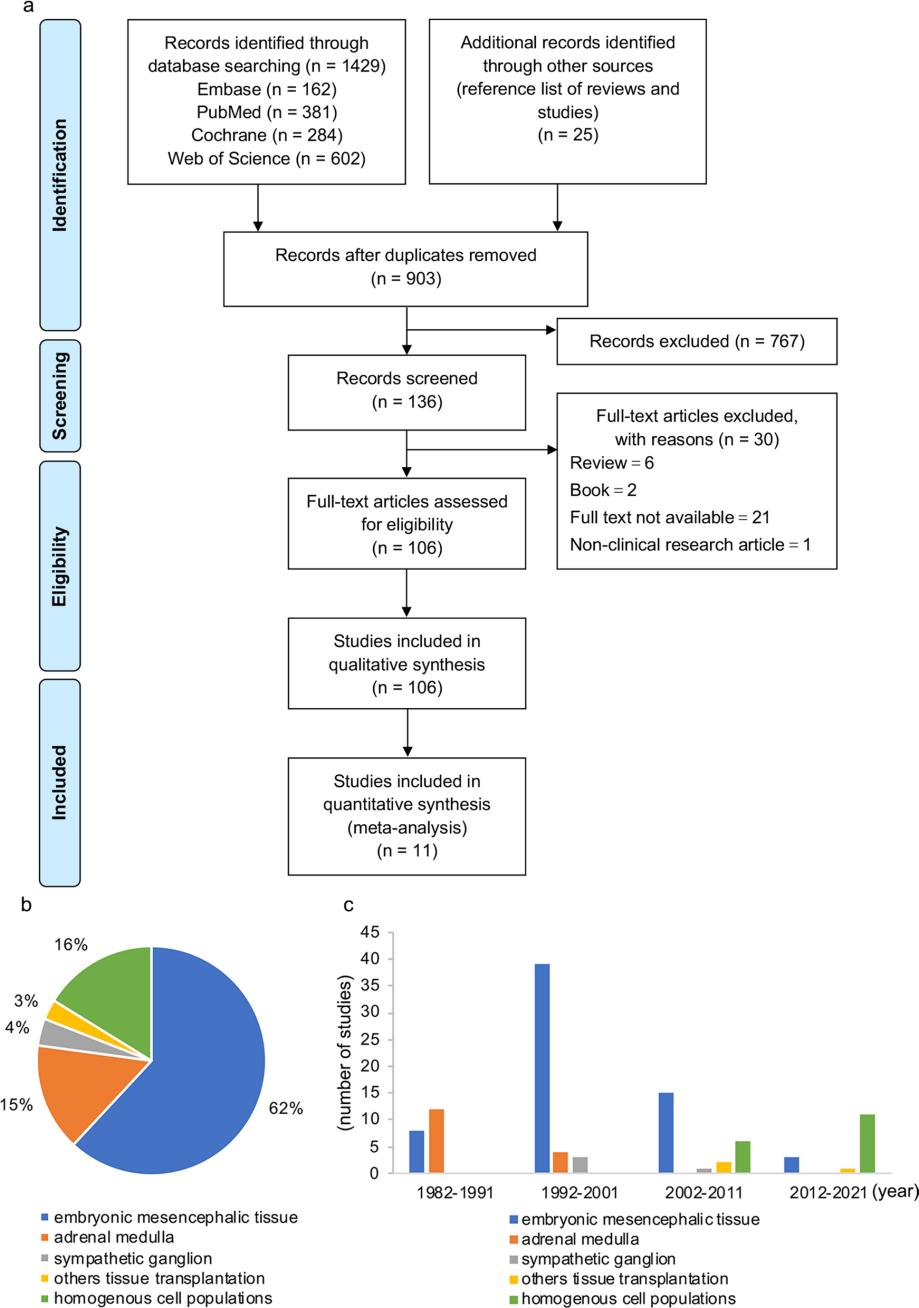
**a** PRISMA flow diagram. **b** Pie chart of the total number of publications on different types of tissue or cell transplantation between 1982 and 2021. **c** Numbers of publications on different types of tissue or cell transplantation in each decade. The numbers of articles on embryonic mesencephalic tissue transplantation published in 1982–1991, 1992–2001, 2002–2011, 2012–2021 are 8, 39, 16 and 3, respectively. Articles reporting adrenal medulla transplantation are 12, 4, 0, and 0. Articles reporting sympathetic ganglion transplantation are 0, 3, 1 and 0. Articles on transplantation of other tissue are 0, 0, 2 and 1. Transplantation of homogenous cell populations are 0, 0, 6 and 11, respectively

Exclusion criteria were: (1) missing or incomplete reporting of efficacy endpoints or sample size; (2) transplantation of mixed or uncharacterized cell populations; (3) case reports. The study selection process is presented in Fig. 1a.

### Data extraction

Data regarding study population, intervention, and outcome were extracted into a standardized form from texts and graphs in each study by the review authors. When only graphic representation was available, values of mean and standard deviation (SD) or standard error (SE) were estimated from high-resolution digital graphs using GetData Graph Digitizer v2.20. Study information including cell source, grafting location, cell dose, sample size, patient age, disease duration, follow-up duration, primary and secondary endpoints, baseline (before transplantation) data, the clinical outcome information, as well as adverse events were collected. Adverse events were defined as an anticipated or unanticipated untoward medical occurrence, unintended disease or injury, or untoward clinical signs (including abnormal laboratory findings) whether or not related to cell transplantation. Neurological function before and after cell transplantation was compared for individual patients to evaluate treatment effects (self-comparison). For RCTs, baseline and outcome data were collected from the treatment groups. SE was converted to SD only when SE was reported.

Outcomes of interest were quantitative neurological scale scores in the ‘on’ or ‘off’ state. The ‘off’ state was defined as a period in which the patients withdrew antiparkinsonian medication for 12 h [10]. The ‘on’ state was at the time of the patients’ peak response to antiparkinsonian medication [10].

### Risk of bias assessment

FW and ZWS independently assessed the risk of bias at the study level of included RCTs and non-RCTs in accordance with the Cochrane Collaboration Guidelines [11]. The risk of bias was assessed as ‘low’, ‘moderate’, ‘high’ or ‘incomplete reporting’ across the following domains: randomization; allocation concealment; blinding of therapists (intervention supervisors); blinding of patients; blinding of outcome assessors; handling of incomplete data (use of intention-to-treat analysis); selective reporting; and multivariate adjustment for potential confounders. Discrepancies in the risk of bias assessment were resolved by discussion among review authors and SL.

### Statistical analysis

The mean difference (MD) or standardized mean difference (SMD) in quantitative neurological scale scores before and after cell-therapy was analyzed to evaluate the treatment effects. Forest plots were created to depict both the pooled MD or SMD along with their 95% confidence intervals (CI). The statistical significance of the pooled effect size of all studies was judged by a Z-test. A *P* value < 0.05 was considered statistically significant. We considered only trials that demonstrated clinical homogeneity to be appropriate for meta-analysis. Potential heterogeneity between studies was initially explored through a visual exploration of the forest plots. A test for statistical heterogeneity (a consequence of clinical or methodological diversity, or both, among trials) was then performed using Cochran’s Q-statistic test (*P* value < 0.1 indicating significance) and *I*^2^ analysis using the following equation:

$$I^2 = \left[ {\left( {{\text{Q}} - {\text{df}}} \right)/{\text{Q}}} \right] \times {1}00\% .$$

in which Q is the Chi^2^ statistic and df is its degrees of freedom. This describes the percentage of variability in effect estimates that is due to heterogeneity rather than sampling error (chance). Values greater than 50% are considered to represent substantial heterogeneity. When values were > 70%, we attempted to interpret the variation. If the value was less than 30%, we presented the overall estimate using a fixed-effect model. If there was evidence of heterogeneity (*I*^2^ > 30%) between trials, we used a random-effect model based on the DerSimonian and Laird method [12]. A leave-one-out sensitivity analysis was performed by iteratively removing one study at a time to confirm whether the findings were driven by any single study. Potential publication bias was evaluated using funnel plots. Review Manager 5.3 was used to complete all statistical calculations.

## Results

### Study characteristics and systematic review of the literature

#### Overview on retrieved records

The initial search returned 903 records, of which 136 were retrieved for full-text review (Fig. 1a). One hundred and six articles were included in the systematic review [4, 13–117]. Eighty-nine articles reported tissue transplantation or transplantation of mixed cell populations, including 66 articles using embryonic mesencephalic tissues (Additional file 9: Table S1) [13–78], 16 articles reporting adrenal medulla tissue transplantation (Additional file 10: Table S2) [79–94], two articles reporting carotid body tissue transplantation [95, 96], four sympathetic ganglion tissue transplantation articles [97–100], and one adipose-derived stromal vascular fraction cell transplantation (Fig. 1b) [101]. Seventeen publications reported transplantation of homogenous cell populations [4, 102–117]. One hundred and four articles explored treatment efficacy, 63 articles reported safety, 92 articles investigated motor symptoms, and 18 articles examined non-motor symptoms. There were 84 articles using allotransplantation, 5 articles on xenotransplantation, and 17 articles on autotransplantation.

#### Changes in predominantly used cell material over time

Predominantly used cell sources for PD treatment changed over time (Fig. 1c). Adrenal medulla tissue transplantation was the most widely studied approach before 1991 (n = 12) and was observed until 2001, but not thereafter. Embryonic mesencephalic tissue transplantation was investigated across all four decades and with most reports published in 1992–2001 (n = 39), gradually decreasing after 2002. Autonomic ganglion tissue transplantation was performed in a few studies between 1992–2011 (n = 3 + 1). Other tissues were investigated by one or two studies only. Treatment with homogenous cell populations became a research focus after 2002 and the most frequently used treatment strategy in the recent decade.

#### Transplantation of allogenic tissues

A total of 297 patients receiving embryonic mesencephalic tissue transplantation were included in this review. These studies investigated different outcomes using a broad range of methods including structural imaging, functional imaging, electrophysiology, biochemical indicators, functional outcome measurements by various scales, and pathological studies by autopsy. Some studies indicated that transplants partially replaced dopaminergic neurons following intra-striatal transplantation, and improved symptoms [41, 46, 51, 75]. Double-blind, sham-controlled clinical trials did not confirm statistically significant benefits from fetal mesencephalic tissue transplantation but revealed adverse events such as GID [20, 23].

#### Transplantation of autologous tissues

The usage of autologous cells is not limited by ethical considerations and avoids severe immune reactions. Autologous cell or tissue transplantation to supply DA was therefore investigated as a potential treatment for PD patients. These autologous DA-secreting cells or tissues included adrenal medulla and carotid body tissues, and sympathetic neurons. In the pioneering work performed by Backlund and collaborators [94], autologous adrenal medulla cells were implanted into the striatum of four patients to provide a local catecholamine source, but the beneficial effects were minimal. In the following 10 years, clinical studies on adrenal medulla transplantation of 148 PD patients yielded similar results and several autopsies demonstrated that the transplanted adrenal cells did not survive in the host brain [118].

The carotid body contains neural crest-derived dopaminergic glomus cells that are similar to the chromaffin cells of the adrenal medulla. These cells function as arterial oxygen sensors and release large amounts of dopamine in response to hypoxia. In addition, glial cell line–derived neurotrophic factor (GDNF) secreted from the carotid body might exert neuroprotective effects for these dopaminergic glomus cells as well as nigrostriatal neurons [119]. A pilot study and a phase I-II blinded clinical study were performed using bilateral intrastriatal transplantation of autologous carotid body cells in patients with advanced-stage PD (n = 6 and 13, respectively) [95, 96]. Functional improvement was seen in five and ten patients, respectively, and no patients developed GID.

Some studies investigated the potential of autologous sympathetic neurons since the ganglion contains not only norepinephrinergic but also dopaminergic cells. Long-term clinical evaluation revealed that unilateral intrastriatal implantation of autologous cervical sympathetic ganglion tissue results in a significant improvement of PD symptoms, particularly akinesia and gait disturbance, and a reduction in the patient’s daily levodopa intake [99]. Following the development of video-guided endoscopic thoracic surgery, it became possible to safely excise three or more ganglia from the thoracic sympathetic trunk in a minimally invasive manner. This option may augment the amount of available tissue, thereby increasing the number of implantation sites. One study endoscopically excised and re-transplanted thoracic sympathetic ganglia in a total of five PD patients [98]. These autografts were found to improve the patients’ performance by reducing the time spent in the off phase. However, there have been no further clinical studies using these cells.

One study investigated intranasal administration of autologous adipose-derived stromal vascular fraction cells in two patients [101]. Both patients exhibited improvements in motor and non-motor functions one and five years after transplantation. There is, however, no clear understanding of the underlying mechanism, and any reported results should be confirmed in future studies.

### Meta-analysis on studies investigating transplantation of homogenous cell populations

Seventeen publications reported transplantation of homogenous cell populations and 11 were eligible for this meta-analysis [102–112]. Two publications were reporting results from one study [112, 113]. Two publications were case reports [4, 117]. Three publications did not report the quantitative data necessary for this analysis. Attempts to contact the corresponding authors failed and these studies were therefore excluded [114–116]. An overview of research protocols and subject characteristics is shown in Table 1.

**Table 1:** Characteristics of the studies and subjects included in the meta-analysis

Study	Patient		Intervention				Follow-up (months)	Outcome	Study type and origin
	N/male	Age (years)	Disease duration (year)	Cell type	Cell count	Graft location			
Schiess 2021 [102]	20/11	66.4 ± 7.0	5.5 ± 1.8	Allogeneic bone marrow mesenchymal stem cells	1, 3, 6, or 10 × 10^6^/kg	Intravenous infusion	3, 6, 12	(1), (3)-(5), (9), (11)	Prospective, single-center, USA
Venkataramana 2012 [103]	8/7	54.6 ± 10.0	8.3 ± 3.8	Allogeneic bone marrow mesenchymal stem cells	2 × 10^6^/kg	Bilateral subventricular zone	12	(1), (8), (11)	Prospective, single-center, India
Boika 2020 [104]	12/7	49.8 ± 12.4	5.7 ± 5.5	Autologous bone marrow mesenchymal stem cells	0.5–2 × 10^6^/kg 5–12.6 × 10^6^/kg	Intravenous infusion Intravenous + intranasal	1, 3	(3), (6), (9)	Prospective, single-center, Belarus
Storch 2012 [105]	7/5	60.6 ± 12.9	10.3 ± 2.9	Autologous bone marrow mesenchymal stem cells	2 × 10^6^	Intrathecal injection + intravenous infusion	1–15	(1), (4), (5)	Retrospective, multi-center, German and Italy
Brazzini 2010 [106]	53/37	61.8 ± 10.7	9.1 ± 5.4	Autologous bone marrow stem cells	NP	Superselective intraarterial (posterior region of the circle of Willis)	1–18	(1), (4)-(6), (11)	Retrospective, single-center, USA
Madrazo 2019 [107]	7/5	54.3 ± 10.9	7.7 ± 5.5	Allogeneic human neural progenitor cells	2 × 10^6^	Bilateral dorsal putamina	12, 24, 48	(1)-(7), (11)-(13)	Prospective, single-center, Mexico
Lige 2016 [108]	21/15	57.3 ± 9.1	NP	Allogeneic human neural precursor cells	3 × 10^7^	Unilateral striatum	7–57	(1)-(9), (11)	Prospective, single-center, China
Sinelnyk 2015 [109]	32/22	47.2 ± 6.7	NP	Allogeneic fetal stem cells	5.46 × 10^6^	Vein and anterior abdominal	6, 12	(5), (6), (8)	Prospective, single-center, USA
Yin 2012 [110]	12/5	66.3 ± 11.9	6.4	Allogeneic human retinal pigment epithelial cells	1 × 10^6^	Unilateral postcommissural putamen	3, 6, 12, 24, 36	(1)-(5), (11), (14)	Prospective, single-center, China
Gross 2011 [111]	35/22	56.4 ± 7.5	NP	Allogeneic human retinal pigment epithelial cells	6.5 × 10^5^	Bilateral postcommissural putamen	12, 24, 36, 48	(2), (3), (5), (9), (11)	RCT, multi-center, USA
Stover 2005 [112]	6/3	52.2	10.2	Allogeneic human retinal pigment epithelial cells	3.25 × 10^5^	Unilateral postcommissural putamen	3, 6, 12, 18, 24	(1)-(3), (9), (10)	Prospective, single-center, USA

*NP* not provided, *RCT* randomized controlled trial, *N* the number of patients

(1) = Unified PD Rating Scale (UPDRS); (2) = UPDRS II; (3) = UPDRS III; (4) = Hoehn and Yahr (H&Y) staging; (5) = Schwab and England scale; (6) = Beck Depression Inventory; (7) = Beck Anxiety Inventory; (8) = Mini-mental State Examination (MMSE); (9) = PDQ-39; (10) = the time in ‘off’ state; (11) = Magnetic Resonance (MR) Imaging; (12) = PET Molecular Imaging ([^18^F]-FDOPA); (13) = PET Molecular Imaging (vesicular monoamine transporter 2, VMAT2); (14) = PET Molecular Imaging (^11^C-β-CFT)

### Risk of bias analysis

The risk of bias assessment is summarized in Table 2. Ten studies were non-RCTs that did not describe the processes of random sequence generation or allocation concealment in sufficient detail. They were considered as incomplete regarding the risk of bias reporting when evaluating selection bias. In most of the studies included for meta-analysis, it was neither practical nor possible to blind the participants or therapists. This was considered a low risk of performance bias for the therapists, but a moderate risk for the participants. Those studies reporting a dropout or loss of follow-up rate higher than 20% were believed to have a high level of attrition bias. Studies were rated as high-risk for detection bias when neither employing intention-to-treat principles in the data analysis nor describing dropouts, nor blinding evaluators to treatment. All other bias assessment domains shown in Table 2 were considered to have a low risk of bias.

**Table 2:** Internal validity of included studies

Study	Prospective design	Multicenter enrollment	Selection bias	Performance bias	Attrition bias	Detection bias	Multivariate adjustment for potential confounders
Schiess 2021 [102]	Yes	No	D	B	A	D	Probably adequate
Venkataramana 2012 [103]	Yes	No	D	B	C	A	Probably adequate
Boika 2020 [104]	Yes	No	D	B	A	D	Probably adequate
Storch 2012 [105]	No	Yes	D	D	C	D	Not reported
Brazzini 2010 [106]	No	No	D	B	A	C	Not reported
Madrazo 2019 [107]	Yes	No	D	B	A	D	Probably adequate
Lige 2016 [108]	Yes	No	D	B	A	D	Not reported
Sinelnyk 2015 [109]	Yes	No	D	B	A	D	Probably adequate
Yin 2012 [110]	Yes	No	D	B	A	A	Probably adequate
Gross 2011 [111]	Yes	Yes	A	A	A	A	Probably adequate
Stover 2005 [112]	Yes	No	D	B	A	D	Probably adequate

Risk of bias is expressed as A = low, B = moderate, C = high, or D = incomplete reporting

### Effects of homogeneous cell populations in PD

#### Disease course and disability

UPDRS (monitoring the disease course and the degree of disability) or UPDRSIII (evaluation of motor function) scores were examined in ‘on’ or ‘off’ state at various post-intervention time points. These follow-ups were different across the nine studies reporting those and varied from 1 to 57 months (last follow-up, Table 1). A total of 210 patients were investigated in the included trials. Meta-analysis was performed on the last follow-up across studies, and at intermediate follow-up time points (3-, 6-, 12-, 24-, and ≥ 36-month follow-ups) when those were reported by the respective studies.

**Fig. 2:**
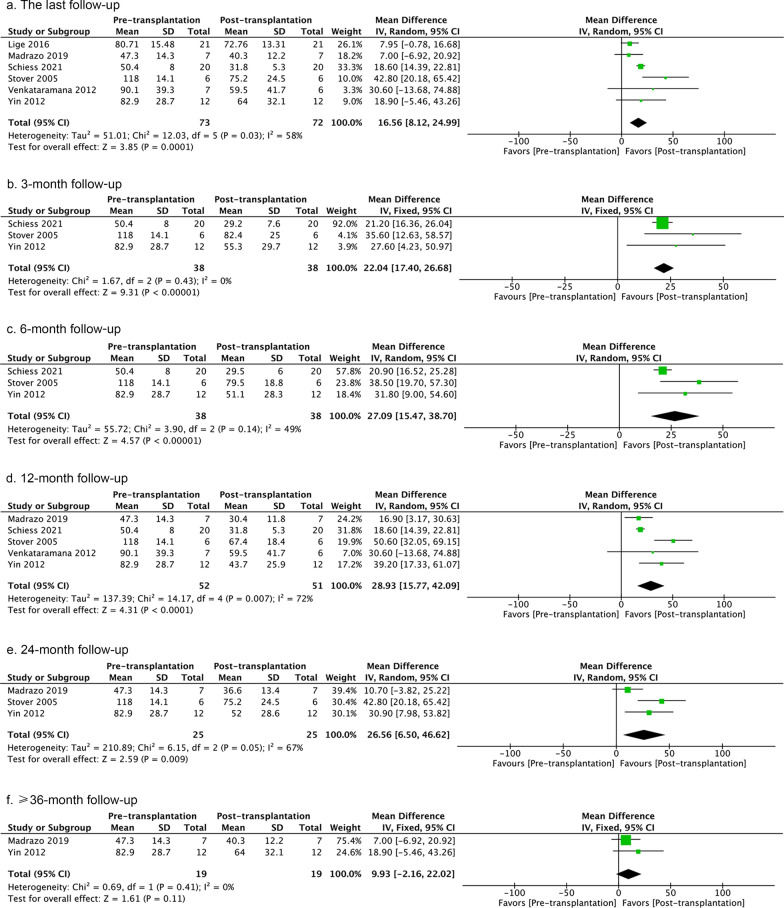
UPDRS scores pre- versus post-transplantation in the ‘off’ state at last follow-up, or 3-, 6-, 12-, 24-, and ≥ 36-month follow-ups. The number of studies included in each analysis are 6, 3, 3, 5, 3, and 2, respectively. If the *I*^2^ value is less than 30%, a fixed-effect model is used. If the *I*^2^ value is greater than 30%, a random-effect model is used. The sizes of the squares represent the weight that each study contributes. The diamond at the bottom represents the overall effect. CI, confidence interval (represented by the lines)

The meta-analysis revealed overall better post- versus pre-treatment function although considerable heterogeneity was evident (Additional file 1: Fig. S1). There was a beneficial effect of homogenous cell-therapy on UPDRS scores in the ‘off’ state at the last follow-up and at 3-, 6-, 12- and 24-month follow-ups, but not at the ≥ 36-month follow-up (Fig. 2). However, the latter was only reported by two studies (Fig. 2). UPDRS scores showed relative homogeneity in the ‘off’ state at 3-, 6-month and ≥ 36-month follow-up analysis (Fig. 2). Moreover, cell treatment improved UPDRS scores in the ‘on’ state at the 12-month follow-up, but not at the last follow-up, or at 24-, ≥ 36-month follow-ups (Additional file 2: Fig. S2). There was no profound heterogeneity among 12-, 24-, ≥ 36-month follow-ups, but at the last follow-up. This might be explained by different transplantation paradigms. For instance, Brazzini et al. infused bone marrow stem cells intraarterially [106], while other studies administered cells directly into the basal ganglia. Removing the study of Brazzini et al. (leaving-one-out analysis) reduced the heterogeneity to 16%, but the overall result remained unchanged (95% CI − 8.95 to 19.03). When analyzing the H&Y scale, we revealed the overall positive effects of cell-therapy at the last assessed timepoints in ‘on’ or ‘off’ state (Additional file 3: Fig. S3). However, there was no change in the levodopa equivalent dose of antiparkinsonian medications after 12 months (*P* = 0.56, *I*^*2*^ = 0%, 95% CI − 103.43 to 191.50).

#### Motor symptoms

**Fig. 3:**
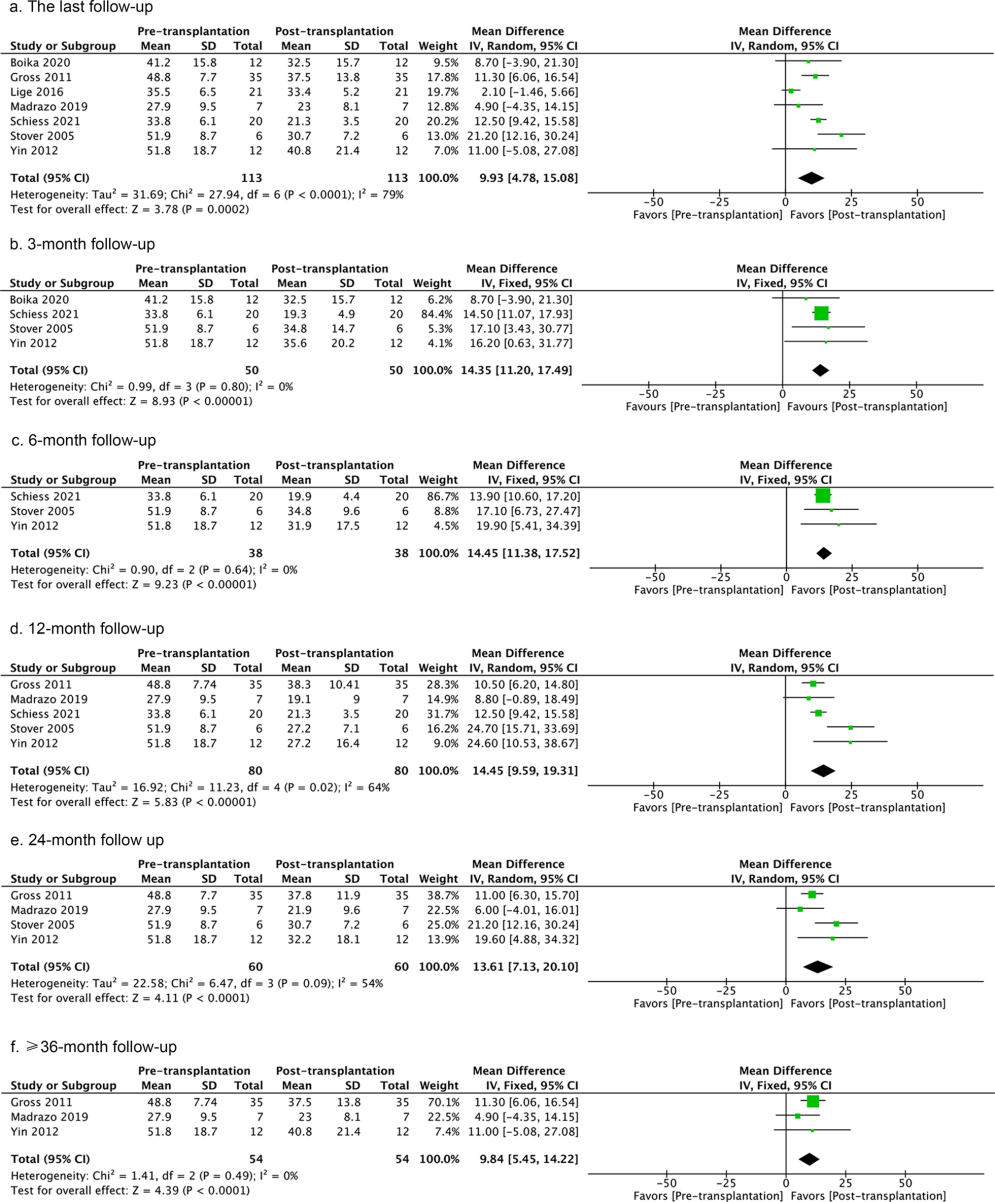
UPDRSIII score pre- versus post-transplantation in the ‘off’ state at last follow-up, or 3-, 6-, 12-, 24-, and ≥ 36-month follow-ups. The number of studies included in each analysis are 7, 4, 3, 5, 4, and 3, respectively. If the *I*^2^ value is less than 30%, a fixed-effect model is used. If the *I*^2^ value is greater than 30%, a random-effect model is used. The sizes of squares represent the weight that each study contributes. The diamond at the bottom represents the overall effect. CI, confidence interval (represented by the lines)

**Fig. 4:**
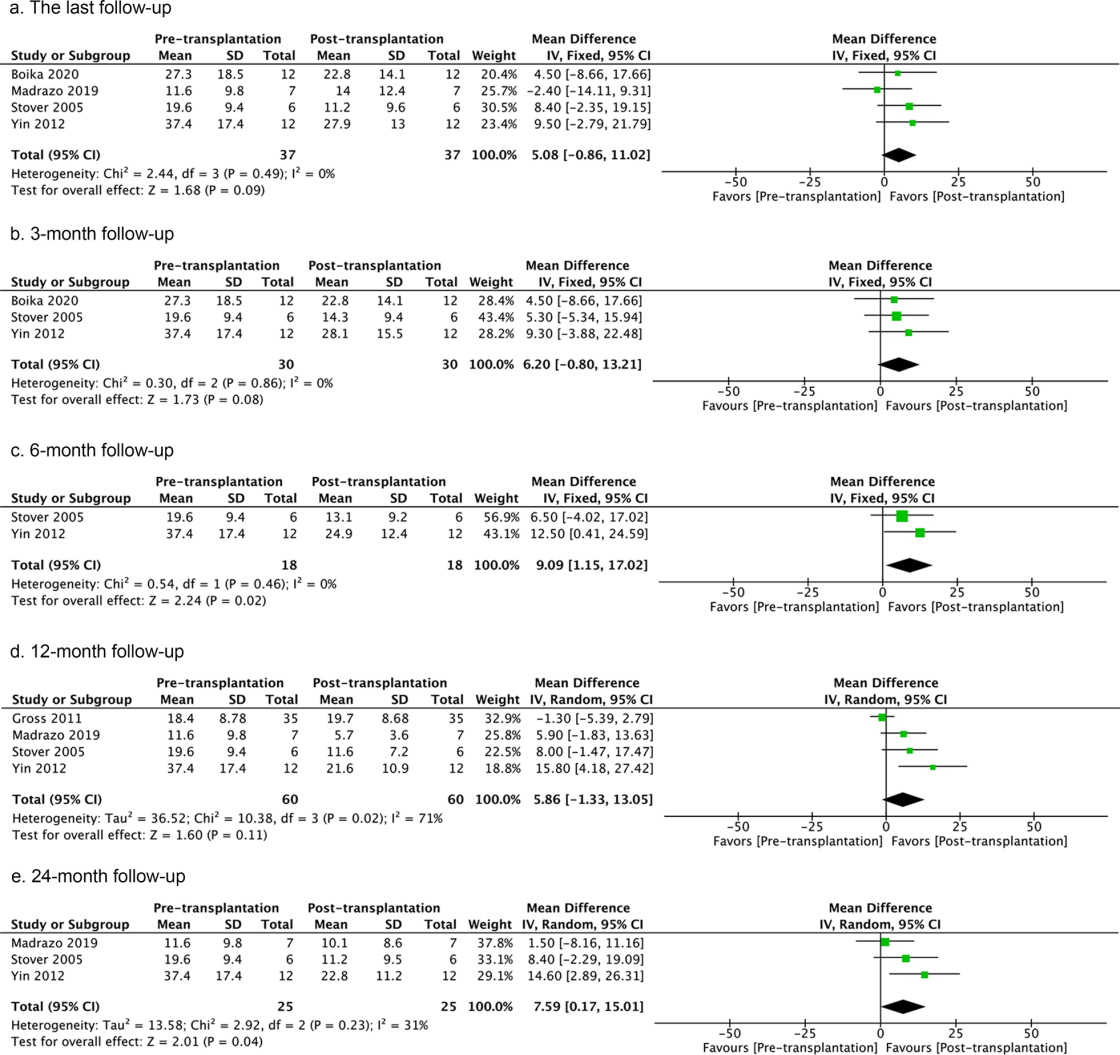
UPDRSIII score pre- versus post-transplantation in the ‘on’ state at last follow-up, or 3-, 6-, 12-, 24-, and ≥ 36-month follow-ups. The number of studies included in each analysis are 4, 3, 2, 4, and 3, respectively. If the *I*^2^ value is less than 30%, a fixed-effect model is used. If the *I*^2^ value is greater than 30%, a random-effect model is used. The sizes of squares represent the weight that each study contributes. The diamond at the bottom represents the overall effect. CI, confidence interval (represented by the lines)

Seven studies measured the effects of homogenous cell-therapy on motor symptoms [102, 104, 107, 108, 110–112]. A random-effect model was used to compare the pre- versus post-treatment UPDRSIII scores in the ‘off’ state at the study last follow-up. The meta-analysis yielded a better outcome after cell treatment, but the heterogeneity was high (Fig. 3). This might be related to the design of the study by Lige et al. who did not use a fixed observation time. Analyzing the UPDRSIII scores after cell treatment in the ‘off’ state at 3-, 6-, 12-, 24-, and ≥ 36-month follow-ups revealed positive effects (Fig. 3, Additional file 4: Fig. S4). Analyzing UPDRSIII scores in the ‘on’ state revealed beneficial effects of cell treatment at the 6- and 24-month follow-ups compared to baseline status, but neither at the last follow-up nor at 3- or 12-month follow-ups (Fig. 4). The inter-study heterogeneity was low at the last follow-up and at 3-, 6-, and 24-month follow-ups (Fig. 4). The 12-month follow-ups showed a high heterogeneity. This can be explained by the study of Gross et al. [111], as its RCT design was different from the other three open-labeled pilot studies. Leaving this study out reduced the *I*^*2*^ value to 0%.

#### Non-motor symptom-depression

Three studies examined the effects of homogenous cell-therapy on non-motor symptoms [106, 107, 109]. The Beck Depression Inventory (BDI) was used to evaluate the degree of depression in patients but did not reveal significant differences after cell treatment. There was considerable heterogeneity between studies probably resulting from diverse transplantation modes (using bilateral basal ganglia transplantation [107], combined intravenous and subcutaneous routes [109], and intra-arterial transplantation [106], respectively) (Additional file 5: Fig. S5).

#### Activities of daily living (ADL)

**Fig. 5:**
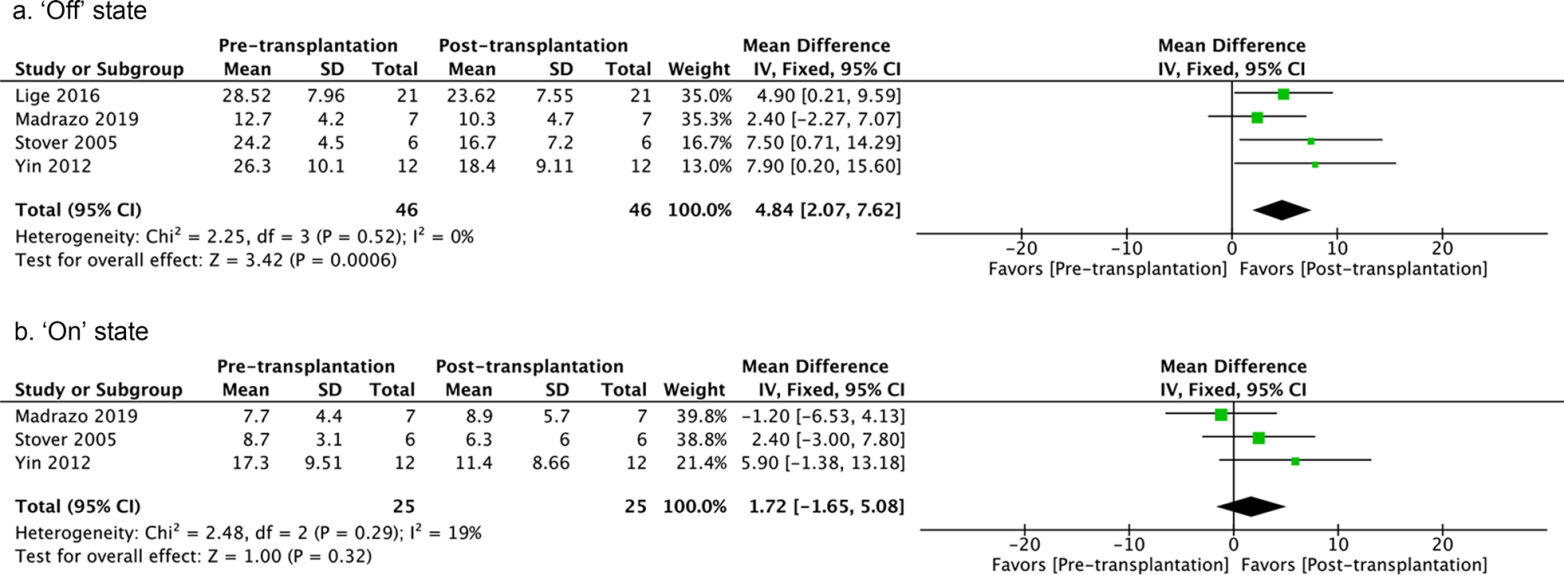
UPDRSII score pre- versus post-transplantation in the ‘off’ and ‘on’ states at the last follow-up. The number of studies included are 4 and 3, respectively. Fixed-effect models are used. The sizes of squares represent the weight that each study contributes. The diamond at the bottom represents the overall effect. CI, confidence interval (represented by the lines)

ADL were assessed using UPDRSII or the Schwab and England score. Four studies examined the UPDRSII scores in the ‘off’ state at the last follow-up [107, 108, 110, 112]. A fixed-effect model revealed a better outcome after homogenous cell-therapy. Three studies assessed UPDRSII scores in the ‘on’ state (all used allogeneic cells) but did not report treatment effects. There was no obvious heterogeneity (Fig. 5) [107, 110, 112].

### Patients potentially benefited from cell-therapy

**Fig. 6:**
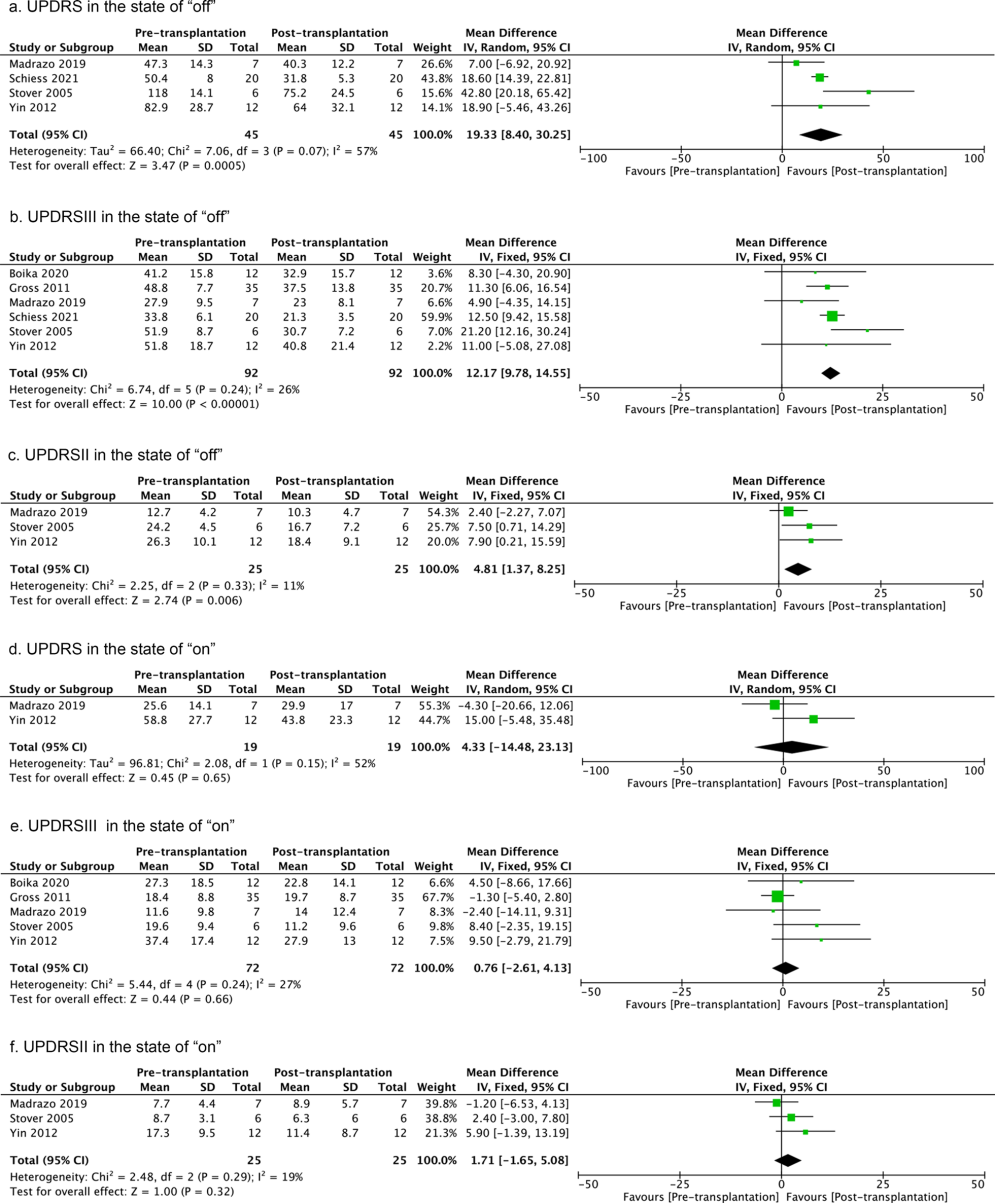
UPDRS, UPDRSIII and UPDRSII scores pre- versus post-transplantation in the ‘off’ and ‘on’ states at the last follow-up with levodopa responders. The number of studies included are 4, 6, 3, 2, 5, and 3, respectively. If the *I*^2^ value is less than 30%, a fixed-effect model is used. If the *I*^2^ value is greater than 30%, a random-effect model is used. The sizes of squares represent the weight that each study contributes. The diamond at the bottom represents the overall effect. CI, confidence interval (represented by the lines)

There was no study investigating whether the effects of cell-therapy are influenced by patient sex. All studies included had equivalent male/female ratio and an average disease course of more than 5 years. The average age was between 47.2 and 66.4 years. Six studies included in the meta-analysis clearly stated that the enrolled patients had positive responses to dopaminergic therapy [102, 104, 107, 110–112]. Five studies did not specify levodopa responsiveness. In the analyses of UPDRS scores in the ‘off’ state, the proportion of levodopa-responsive patients was 100%, 100%, 86.5%, 100% and 100% at 3-, 6-, 12-, 24-, and ≥ 36-month follow-ups and 61.6% at last follow up, respectively (Fig. 2). The fraction of levodopa-responsive patients was 76%, 100%, 100%, 100%, 100% at 3-, 6-, 12-, 24-, and ≥ 36-month follow-ups, and 81.4% at last follow-up, in the analyses of UPDRSIII scores in the ‘off’ state, respectively (Fig. 3). The patients who were responsive to dopaminergic therapy showed functional improvements on UPDRS, UPDRSIII, and UPDRSII scores in the ‘off’ state at the last follow-up, but not in the ‘on’ state (Fig. 6).

### Impact of cell immunogenicity and cell type on outcome

Eight studies used allogeneic, and three studies used autologous cells for transplantation. Allogeneic cells (neural progenitor cells, fetal stem cells, retinal pigment epithelial cells, and bone marrow mesenchymal stem cells) showed beneficial effects on UPDRS, UPDRSIII, and UPDRSII scores in the ‘off’ state at the last follow-up, but not in the ‘on’ state (Additional file 6: Fig. S6) [102, 103, 107, 108, 110–112]. There were considerable heterogeneities in the UPDRSIII score analyses in the ‘off’ state, which might be explained by one study not defining fixed observation time points (last follow-up ranged from 7–57 months)[108]. Removing this study reduced the *I*^*2*^ value to 37% but did not change the overall result. When autografts (mesenchymal stem cells that cannot differentiate into neural cells) were used, no beneficial effect was observed on H&Y scores in ‘off’ or ‘on’ state (Additional file 7: Fig. S7) [105, 106]. Even though homogenous cell-therapy in general and allogeneic cells in particular showed positive effects on motor function in the ‘off’ state, autologous cell transplantation did not show such effects.

**Fig. 7:**
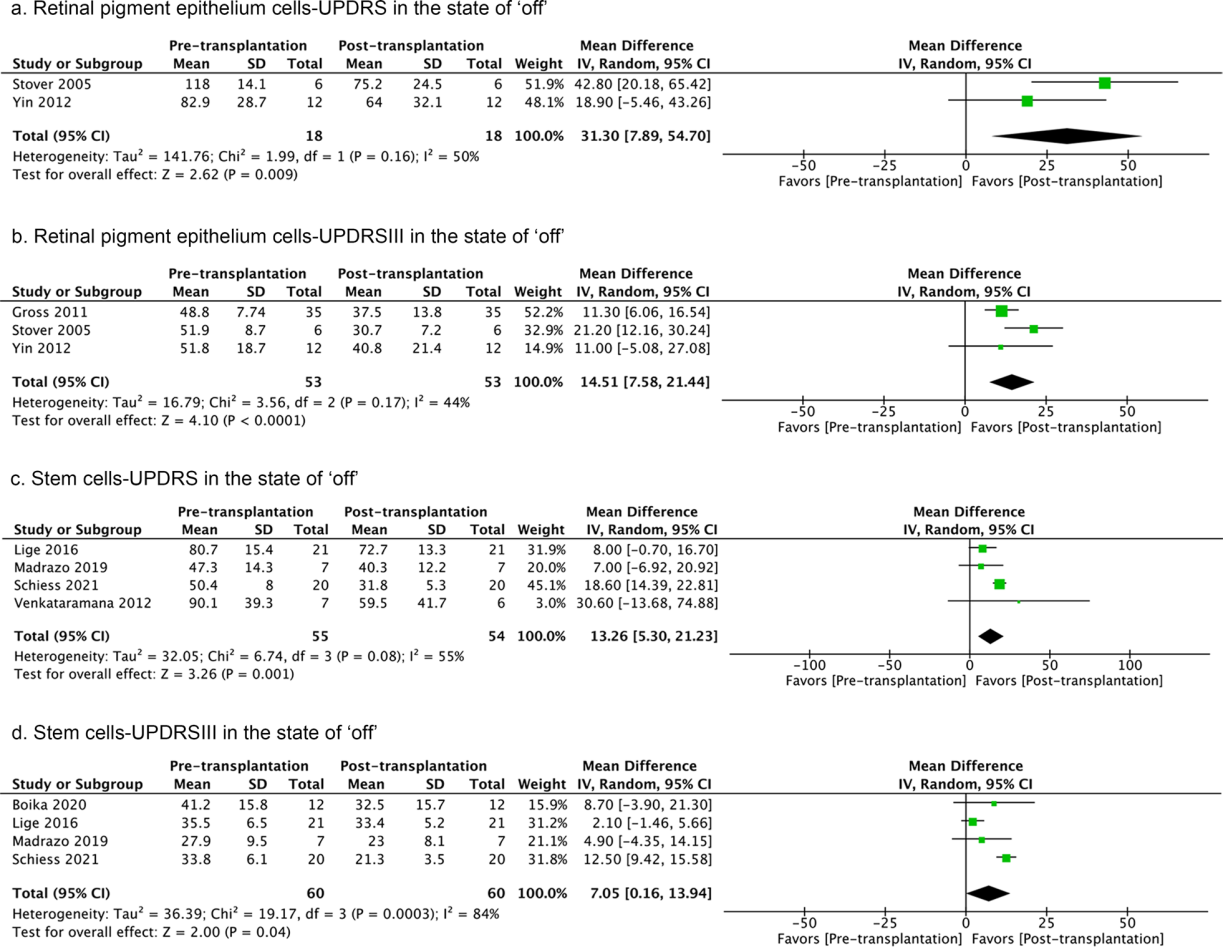
UPDRS and UPDRSIII scores pre- versus post-transplantation in the ‘off’ state at the last follow-ups after retinal pigment epithelium cell and stem/progenitor cell treatment. The number of studies included are 2, 3, 4, and 4, respectively. Random-effect models are used. The sizes of squares represent the weight that each study contributes. The diamond at the bottom represents the overall effect. CI, confidence interval (represented by the lines)

Several types of cells were transplanted including neural progenitor cells (n = 2), fetal stem cells (n = 1), bone marrow mesenchymal stem cells (n = 4), other bone marrow stem cells (including exact cell type not specified, n = 1), and retinal pigment epithelial cells (n = 3). UPDRS or UPDRSIII assessments in the ‘off’ states at the last follow-up revealed better outcomes after retinal pigment epithelium cell and stem/progenitor cell treatment (Fig. 7). The heterogeneity was low in retinal pigment epithelial cell studies. However, the UPDRSIII analysis of stem/progenitor cell-therapy revealed high heterogeneity, potentially due to the study of Lige et al. not defining fixed observation time points [108]. Removing this study reduced the *I*^*2*^ value to 21%.

### Transplantation route

**Fig. 8:**
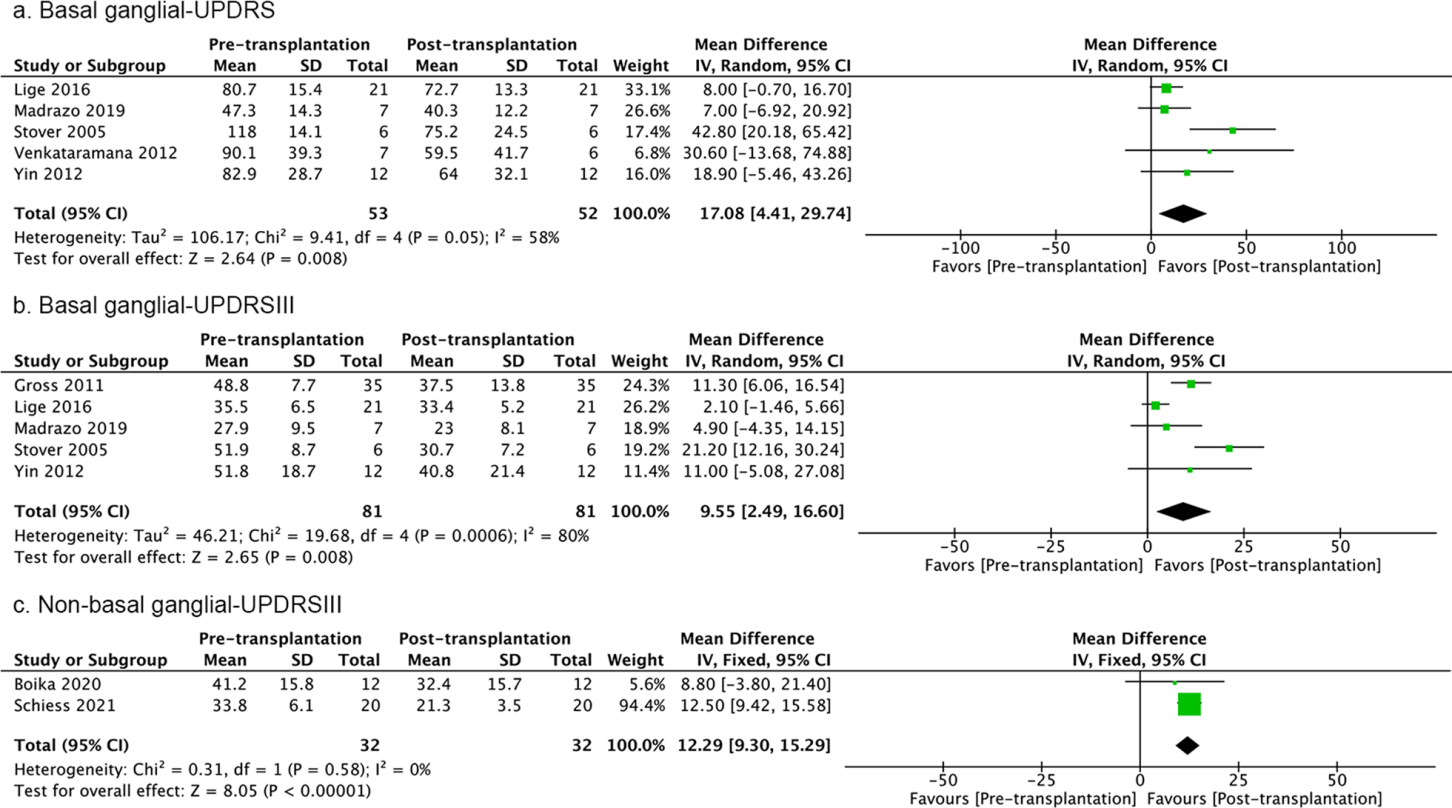
UPDRS and UPDRSIII scores pre- versus post-transplantation in the ‘off’ state at the last follow-ups after basal ganglia and non-basal ganglia transplantation. The number of studies included are 5, 5, and 2, respectively. If the *I*^2^ value is less than 30%, a fixed-effect model is used. If the *I*^2^ value is greater than 30%, a random-effect model is used. The sizes of squares represent the weight that each study contributes. The diamond at the bottom represents the overall effect. CI, confidence interval (represented by the lines)

Among the 11 studies included in this meta-analysis, six studies performed intraparenchymal transplantation into the basal ganglia (Table 1) [103, 107, 108, 110–112]. Unilateral and bilateral intraparenchymal transplantation was performed in three studies each. One study investigated intravenous infusion of allogeneic bone marrow-derived mesenchymal stem cells [102]. One study transplanted autologous mesenchymal stem cells through intravenous or tandem (intranasal + intravenous) injections [104]. One study combined intravenous and subcutaneous transplantation of fetal stem cells [109]. One study injected autologous bone marrow mesenchymal stem cells via intrathecal and intravenous injection [105]. One study infused bone marrow stem cells using a superselective intraarterial approach to the posterior region of the circle of Willis [106]. Basal ganglia transplantation resulted in beneficial effects on both UPDRS and UPDRSIII scores in ‘off’ state at the last follow-ups. Non-basal ganglia transplantation improved UPDRSIII scores. However, the *I*^*2*^ value for UPDRSIII scores were high for transplantation into basal ganglia, which might again be explained by the study of Lige et al. (Fig. 8) [108].

### Cell doses

The cell doses used for transplantation were between 1 and 10 × 10^6^/kg in the ten studies investigated. In one study, four doses (1, 3, 6, or 10 × 10^6^/kg) of allogeneic bone marrow-derived mesenchymal stem cells were administered intravenously to investigate a potential dose-dependent efficacy [102]. The results showed that all doses showed effects on motor symptoms in the ‘off’ state. However, the highest dose achieved the maximum absolute improvement at the 52 weeks follow-up and reduced the UPDRS motor and total scores in the ‘off’ state. Therefore, the included studies suggested that cell doses between one to ten million were all effective.

### Imaging readouts

Seven included studies applied magnetic resonance (MR) imaging for outcome evaluation [102, 103, 106–108, 110, 111], among which four [107, 108, 110, 111] investigated safety endpoints including inflammatory responses, tumor formation, bleeding, and edema after cell transplantation. Three other studies [102, 103, 106] used MR spectroscopy, MR perfusion, and MR tractography for efficacy evaluation. In one study, MR spectroscopy revealed a significant increase of the mean n**-**acetylaspartate/creatine ratio in basal ganglia after transplantation [106]. One study showed MR perfusion increased overall from baseline to 24 weeks post infusion in all basal ganglia structures [102]. Another study reported a statistically non-significant trend of improvement in fractional anisotropic (FA) values of MR tractography in the genu and the cerebral peduncles steadily over a period of 12 months after transplantation [103]. Two studies employed positron emission tomography (PET) imaging to evaluate the efficacy [107, 110]. The radiopharmaceuticals included FDOPA, DTBZ, and ^11^C-β-CFT. FDOPA and DTBZ imaging showed a statistically non-significant trend toward enhanced midbrain dopaminergic activity at one year after grafting in one study [107]. The other study showed a statistically non-significant trend towards increased dopamine release in ^11^C-β-CFT PET imaging during the first 6 months after transplantation [110]. These studies suggested that cell-therapy partially replaced dopaminergic neurons. Due to the heterogeneity in imaging methodology, the limited number of studies and overall small sample sizes, however, prevented a meaningful meta-analysis of the imaging readouts regarding efficacy.

### Adverse events of homogenous cell transplantation

The reports for adverse events of homogenous cell-therapy for PD are listed in Table 3. No tumor formation or severe immune rejections were observed. Two trials reported GID [102, 111]. There were other adverse events including surgical injury and complications, such as phlebitis and hematoma. Psychonosema was noted such as hallucination or disturbance in attention.

**Table 3:** Adverse events and immunosuppression

Study	Sample size	Adverse events				Immunosuppression
		Tumor	Immune rejection	GID	Others	
Schiess 2021 [102]	20	No	No	4 patients (possibly related)	Phlebitis, hematoma, hypertension, nausea, headache, lymphocytosis	Not performed
Venkataramana 2012 [103]	8	No	No	No	No	Not performed
Boika 2020 [104]	12	NP	NP	NP	NP	Not performed
Storch 2012 [105]	7	No	No	NP	NP	NP
Brazzini 2010 [106]	53	No	No	No	No	Not performed
Madrazo 2019 [107]	7	No	No	No	No	Cyclosporine A for 40 days
Lige 2016 [108]	21	No	No	No	No	Not performed
Sinelnyk 2015 [109]	32	No	No	No	No	Not performed
Yin 2012 [110]	12	No	No	No	No	Not performed
Gross 2011 [111]	35	No	No	1 patient	Disturbance in attention, hallucination	Not performed
Stover 2005 [112]	6	No	No	No	Hallucination	Not performed

*GID* graft-induced dyskinesia, *NP* not provided

### Sensitivity analysis

Sensitivity analyses were performed to evaluate the robustness of the estimated pooled effect size for UPDRS, UPDRSIII, UPDRSII scores and non-motor symptoms. The pooled effect was stable for UPDRSIII and UPDRSII in the ‘off’ state and non-motor symptoms, indicating that these results were not driven by any single study. However, when either the study by Brazzini et al. [106] or the one by Lige et al. [108] was removed, statistical significance was lost for the pooled effect size of homogenous cell-therapy on H&Y scores in the ‘on’ or ‘off’ state at the last follow-up. On the contrary, when the study of Madrazo et al. [107] was removed, cell-therapy became beneficial for UPDRS and UPDRSIII scores in the ‘on’ state at the last follow-up. Removing the study of Gross et al. [111] also resulted in the detection of a cell treatment effect on UPDRSIII scores in the ‘on’ state at the 12-month follow-up.

### Publication bias

Funnel plots were plotted for the meta-analysis including more than 5 studies (Additional file 8: Fig. S8). These plots were symmetrical and evenly distributed, and few effects fell outside the 99% CI, suggesting that the present meta-analyses were not substantially affected by publication bias.

## Discussion

### Tissue transplantation

Intracerebral grafting of fetal mesencephalic tissue, which is rich in dopaminergic neuroblasts, was first reported in 1979, ameliorating the symptoms of experimental PD rats [120, 121]. Thereafter, about 400 PD patients were grafted with human fetal mesencephalic tissue in the 1980s–1990s. Fetal tissue grafts have survived over two decades in some patients despite ongoing PD pathology [122]. In addition, several trials showed engraftment of fetal tissue with wide outgrowth and robust innervation of the host striatum by donor-derived DA neurons [54, 56, 58, 66, 123, 124]. However, due to GID, fetal tissue transplantation was abandoned. The overall discouraging results may be partly related to differences between studies in cell sources, preparation, and transplantation paradigms [23, 124]. In addition, multiple fetal donors (typically 3–5) were pooled to obtain sufficient numbers of cells for one patient. This may contribute to the heterogeneity of outcomes and may indicate a lack of material for widespread clinical usage. Ethical arguments also limit fetal tissue transplantation. Therefore, transplantation of human fetal mesencephalic tissue is very unlikely to be developed into a routine treatment for PD patients.

Autologous adrenal medulla and carotid body tissues, and sympathetic neurons were explored as PD treatments because these can either secret DA or exert neurotrophic effects, but their precise therapeutic mechanism is uncertain [95–100]. Tissue transplantation was less investigated in the recent decade. Lately, the concept experienced a renaissance due to advances in regenerative medicine and tissue engineering, using optimized grafting and defined immunosuppression protocols [2]. Successful *in-vitro* differentiation of embryonic stem cells [125–127] or induced pluripotent stem cells [4] towards a midbrain dopaminergic fate may allow the development of cell-therapies for PD while avoiding many practical and ethical concerns regarding tissue transplantation, although there are still many challenges in translating *in-vitro* success to *in-vivo* applications, and potential ethical concerns surrounding embryonic stem cells usage. What remains is the need for cell transplants that can not only functionally integrate but survive in the host brain over long periods.

### Therapeutic effects of homogenous cell-therapy on PD

To the best of our knowledge, this is the most comprehensive meta-analysis of clinical trials on cell treatments for PD to date. Both cell origin and the site of cell transplantation varied considerably across the studies. Most transplantations (6 out of 11) were performed into basal ganglia uni- or bilaterally. Follow-up time ranged from 1 to 57 months. The key finding from our meta-analysis is that homogenous cell transplantation significantly improves clinical outcomes in PD patients regarding overall disease severity, motor symptoms, and ADL in the ‘off’ state.

The main outcome measurement in our meta-analysis was based on the UPDRS score which is believed to be less susceptible to observer bias than other scores [128]. Therefore, it is less likely that the clinical improvements observed can be solely attributed to observer bias. Our findings suggest that the investigated cell treatments have a robust effect on the ‘off’ state at the 3-, 6-, 12-, 24-, and even ≥ 36-month follow-ups for motor symptoms. There was indication that the magnitude and duration of functional improvement induced by dopaminergic grafts depend on patient selection, with good preoperative response to L-dopa predicting good response to the graft [129, 130]. In this meta-analysis most of the patients included were responsive to dopaminergic therapy, and those patients may also be responsive to cell-therapy. Patients with DA neuron loss restricted to the caudate and putamen are more likely to experience long-term benefits from dopaminergic grafts placed in these areas [14, 25, 129, 130]. In contrast, long-lasting beneficial outcomes in PD patients with more widespread DA neuron loss are less likely [14].

Most of the trials in our meta-analysis included PD patients with a good response to L-dopa. This may explain why UPDRS and UPDRSIII at the ‘on’ state did not improve much, as the combination of both treatments would require a significant additional effect that may not be detected with the overall limited numbers of patients investigated. No difference between neurological function pre- and post-transplantation was found in the UPDRS score in the ‘off’ state at ≥ 36 months. Graft function may be compromised by delayed immune reactions, previously characterized by microglial infiltration into the graft [23]. However, UPDRSIII scores in the ‘off’ state at ≥ 36 months provide preliminary evidence that the cell graft was still functional, but more rigorous RCTs and long-term follow-up studies, especially those ≥ 36 months are needed to confirm this. Those should include tailored assessment of graft functionality, for instance by sophisticated brain imaging.

Only few clinical trials investigating homogenous cell-therapy for PD have focused on the management of non-motor symptoms: four articles investigated cognition [102, 103, 107, 109], four articles reported depression [104, 106, 107, 109], one studied anxiety article [107], and two examined sleep-disorder [104, 109]. Although a significant decrease of non-motor symptoms and depression, as well as an improvement in objective parameters of sleep quality, were reported in PD patients after cell treatment in single studies [109], we could not confirm these findings in our meta-analysis. Several factors could have contributed to this. Firstly, non-motor symptoms may originate from degeneration outside the striatum or in non-dopaminergic systems that may be difficult to target with cell-therapy. Secondly, the cell grafts investigated may simply lack the ability to counter these symptoms. Third and most importantly, the relatively high inter-study heterogeneity regarding cell type and source, transplantation site, and other aspects may just have ‘masked’ minor yet clinically meaningful effects on these endpoints. Therefore, it is crucial to scrutinize non-motor symptoms in future investigations. In summary, the overall positive impact on ADL parameters observed in our meta-analysis may primarily originate from motor symptom improvements. However, overall results should be interpreted with caution as the overall number of available and included studies is relatively low.

### Effects of different cell sources and transplantation modes on efficacy

Most of the included studies (n = 8) transplanted allogeneic cells for PD patients and exhibited robust beneficial effects on UPDRS, UPDRSIII, and UPDRSII scores in the ‘off’ state. However, autografts were ineffective in symptoms examined by H&Y score changes in ‘off’ or ‘on’ states as there were not sufficient autograft transplantation studies to be combined to evaluate the UPDRS changes. The three articles evaluating autografts all used bone marrow mesenchymal stem cells [104–106], which may not differentiate into neural tissue. However, they may exert beneficial immunomodulative and neuroprotective effects. Moreover, six out of the eight articles evaluating allogeneic cells used neural progenitor cells, fetal stem cells, or retinal pigment epithelial cells. Those might be able to differentiate into neuronal cells. Thus, allogeneic cells and autologous cells likely have different mechanisms of action. Therefore, it is rational to speculate that the overall positive effects of homogenous cell-therapy for PD patients in our meta-analysis were mainly due to allogeneic cell transplantation studies and that allogeneic cells may be a better option for PD treatment, particularly, retinal pigment epithelium cell and stem/progenitor cell. Besides, allogeneic cells have some logistical advantages as they can be obtained and prepared in advance and under standardized conditions. They might also be advantageous in inherited PD. However, when using allogeneic cells, the immunological barrier represents a formidable obstacle for the transplanted cells to survive and execute therapeutic effects relying on differentiation and functional integration. Fortunately, with the development of modern immunosuppressants, graft survival and side effects have been greatly improved [131].

Unexpectedly, we observed that transplantation outside basal ganglia was also effective to improve motor function in PD patients. In these two studies, the intravenously infused bone marrow mesenchymal stem cells were likely to improve PD symptoms through immunomodulatory mechanisms, such as decreasing inflammatory cytokine production, reducing microglial activation and a-synuclein oligomerization [102, 104]. This observation may be clinically relevant because such transplantation, in particular systemic cell delivery, may not only be safer and easier to perform, but also less expensive and time-consuming. However, this result is based on a limited number of studies and thus will require confirmation, and the likelihood of immunological consequences is far greater after systematic cell delivery.

Other factors as, for instance, gender, age, and disease courses of the patients may also act as confounding factors. However, due to the lack of available raw data, we were unable to analyze their impact on reported functional outcomes after cell-therapy.

### Adverse events of homogenous cell-therapy

No tumor formation or severe immune rejections were reported in the included studies, but one trial reported a case of GID, and another trial reported four cases of possibly GID. Off-state GID was a relatively frequent adverse event after human fetal mesencephalic tissue transplantation. The interpretation of this phenomenon is difficult. Modeling studies suggest that some form of L-DOPA-induced postsynaptic supersensitivity, established before transplantation, may play a role [132, 133]. Moreover, small, intracerebral transplants may be more prone to cause GID by forming ‘hot-spots’ of DA release, while the surrounding striatum remains supersensitive [133, 134]. Finally, a potential role of excessive serotonin innervation has been discussed [8, 135, 136]. Fetal mesencephalic tissue often used for transplantation also contains serotonergic neurons, and studies on 6-hydroxydopamine-induced PD models suggested that these could exacerbate dyskinesia induced by L-DOPA [135]. Clinical research suggested that a non-optimal ratio between serotonergic and dopaminergic neurons (or their progenitors) in grafts causes GID [136–138]. The relatively low incidence of GID in the studies included in our meta-analysis may be related to patient selection, improvement of surgical methods, and higher homogeneity of transplanted cells. Other adverse events such as surgical complications (phlebitis and hematoma) and psychonosema were generally rare. However, two included studies did not provide comprehensive adverse effect reports, which limit the understanding of potential risks associated with the intervention. Due to the inconsistency in reporting of adverse events, we were also unable to compare the safety profiles of different interventions. A thorough and robust safety analysis is imperative for future clinical trials.

### Quality of evidence and limitations

Despite the generally encouraging results of our meta-analysis, it is important to keep in mind that most of the included studies were open-label, single-center trials, with outcome data not reported or inadequately described in some studies. Moreover, insufficient information on disease duration in some studies limits the understanding of how the disease stage could affect the treatment outcomes and impact the quality and reliability of the analysis. Although blinding of the participants and therapists was not possible, outcome assessors can be blinded. Nevertheless, a relatively large proportion of studies (n = 5, 41.7%) did not report blinding of outcome assessors. Thus, the results may still contain observer bias.

Another major limitation is that our meta-analysis cannot provide a thorough perspective on how cell-therapies for PD may be improved further. The main reasons are the small number of studies and the overall heterogeneity of cell and tissue types being used. While there is an overall positive effect of cell-based treatments, any kind of optimal approach cannot be identified from this relatively small dataset. Moreover, we can only speculate why systemic cell administration was effective or why overall best effects were obtained with allogeneic cells, and both findings may appear counter-intuitive. A combination of thorough preclinical and clinical research is required to solve these questions. Mechanistic investigations in relevant animal models should identify the most effective cell types and transplantation paradigms while multicenter, large-scale, and double-blinded RCTs are needed to verify the encouraging yet preliminary results of our meta-analysis. Alternative solutions, such as pharmacological therapy and deep brain stimulation, should also be considered in conjunction with cell-based therapies.

## Conclusion

According to this meta-analysis, cell therapy was effective for improving disease severity and motor symptoms while also improving ADL in the ‘off’ state of PD patients, especially in levodopa responders. Allogenic cells exerted beneficial effects on these parameters, but autografts did not. Transplantation of cells to areas outside the basal ganglia, including system transplantation of cells, was able to induce therapeutic benefits. Some trials reported adverse events potentially related to the surgical procedure. One confirmed and four possible cases of GID were reported in two trials included in meta-analysis. Therefore, our results suggest modest yet clinically meaningful cell therapy effects in patients with PD although definitive evidence must be provided by future double-blinded large-scale RCTs. These should also monitor the long-term safety of cell-based interventions for PD while the optimal cell population and route of transplantation need to be defined. Cell-therapies in PD are not a stand-alone treatment but must always be considered in combination with established therapies.

### Supplementary Information

**Additional file 1: Fig. S1**. UPDRS or UPDRSIII scores pre- versus post-transplantation in ‘on’ or ‘off’ state at the last follow-up. Nine studies are included. Random-effect model is used. The sizes of squares represent the weight that each study contributes. The diamond at the bottom represents the overall effect. CI = confidence interval (represented by the lines).

**Additional file 2: Fig. S2**. UPDRS score pre- versus post-transplantation in the ‘on’ state at the last follow-up, or at 12-, 24-, and ≥ 36-month follow-ups. The number of studies included are 4, 3, 2, and 2, respectively. If the *I*^2^ value is less than 30%, a fixed-effect model is used. If the *I*^2^ value is greater than 30%, a random-effect model is used. The sizes of squares represent the weight that each study contributes. The diamond at the bottom represents the overall effect. CI = confidence interval (represented by the lines).

**Additional file 3: Fig. S3**. H-Y score pre- versus post-transplantation in the ‘on’ or ‘off’ states at the last follow-up. Four studies are included. Random-effect model is used. The sizes of squares represent the weight that each study contributes. The diamond at the bottom represents the overall effect. CI = confidence interval (represented by the lines).

**Additional file 4: Fig. S4**. UPDRSIII score pre- versus post-transplantation in the ‘off’ state at 48-month follow-up. Two studies are included. Fixed-effect model is used. The sizes of squares represent the weight that each study contributes. The diamond at the bottom represents the overall effect. CI = confidence interval (represented by the lines).

**Additional file 5: Fig. S5**. Beck Depression inventory score pre- versus post-transplantation in the ‘on’ or ‘off’ states at the last follow-up. Three studies are included. Random-effect model is used. The sizes of squares represent the weight that each study contributes. The diamond at the bottom represents the overall effect. CI = confidence interval (represented by the lines).

**Additional file 6: Fig. S6**. UPDRS, UPDRSIII and UPDRSII scores pre- versus post-transplantation in the ‘off’ and ‘on’ states at the last follow-ups after allogeneic cell treatment. The number of studies included are 6, 6, 4, 3, 3, and 3, respectively. If the *I*^2^ value is less than 30%, a fixed-effect model is used. If the *I*^2^ value is greater than 30%, a random-effect model is used. The sizes of squares represent the weight that each study contributes. The diamond at the bottom represents the overall effect. CI = confidence interval (represented by the lines).

**Additional file 7: Fig. S7**. H-Y score pre- versus post-transplantation in the ‘on’ or ‘off’ states at the last follow-up after autologous cell treatment. Two studies are included. Random-effect model is used. The sizes of squares represent the weight that each study contributes. The diamond at the bottom represents the overall effect. CI = confidence interval (represented by the lines).

**Additional file 8: Fig. S8**. Funnel plots assessing potential publication bias on homogeneous cell transplantation in PD treatment. (a) UPDRS or UPDRSIII scores pre- versus post-transplantation in ‘on’ or ‘off’ state at the last follow-up. (b) UPDRS score pre- versus post-transplantation in the ‘off’ state at last follow-up. (c) UPDRSIII score pre- versus post-transplantation in the ‘off’ state at last follow-up. (d) UPDRSIII scores pre- versus post-transplantation in the ‘off’ states at the last follow-up with levodopa responders. (e) UPDRS scores pre- versus post-transplantation in the ‘off’ states after allogeneic cell treatment. (f) UPDRSIII scores pre- versus post-transplantation in the ‘off’ states at the last follow-ups after allogeneic cell treatment. Each dot represents a single study. The dashed vertical line represents the pooled effect size. The dashed diagonal lines represent 95% confidence limits around the pooled effect size for each standard error on the vertical axis, and are only provided in plots when fixed effect models were used.

**Additional file 9: Table S1**. Fetal mesencephalic tissue transplantation: characteristics of the studies and subjects.

**Additional file 10: Table S2**. Adrenal medulla transplantation: characteristics of the studies and subjects.

## Data Availability

The data that support the findings of this study are available from the corresponding author upon reasonable request.

## Abbreviations

ADL: Activities of daily living
BDI: Beck Depression Inventory
CI: Confidence interval
DA: Dopamine
FA: Fractional anisotropic
GDNF: Glial cell line-derived neurotrophic factor
GID: Graft-induced dyskinesia
H&Y: Hoehn and Yahr Staging Scale
MD: Mean difference
MMSE: Mini-mental State Examination
MR: Magnetic Resonance
PD: Parkinson’s disease
PET: Positron emission tomography
RCT: Randomized controlled trial
SD: Standard deviation
SE: Standard error
SMD: Standardized mean difference
UPDRS: Unified Parkinson Disease Rating Scale
