# Supplementary figures and images for: Cell-therapy for Parkinson’s disease: a systematic review and meta-analysis
Source: J Transl Med. 2023 Sep 7;21:601. doi: 10.1186/s12967-023-04484-x (PMC10483810; doi:10.1186/s12967-023-04484-x)

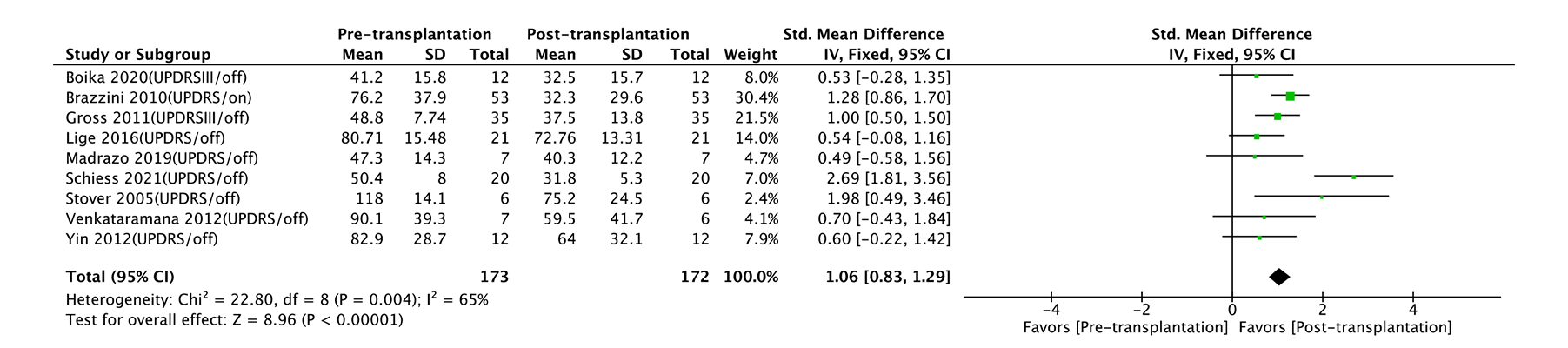

Supplement: Supplementary file 1 — Additional file 1: Fig. S1. UPDRS or UPDRSIII scores pre- versus post-transplantation in ‘on’ or ‘off’ state at the last follow-up. Nine studies are included. Random-effect model is used. The sizes of squares represent the weight that each study contributes. The diamond at the bottom represents the overall effect. CI = confidence interval (represented by the lines). [file 12967_2023_4484_MOESM1_ESM.tif]

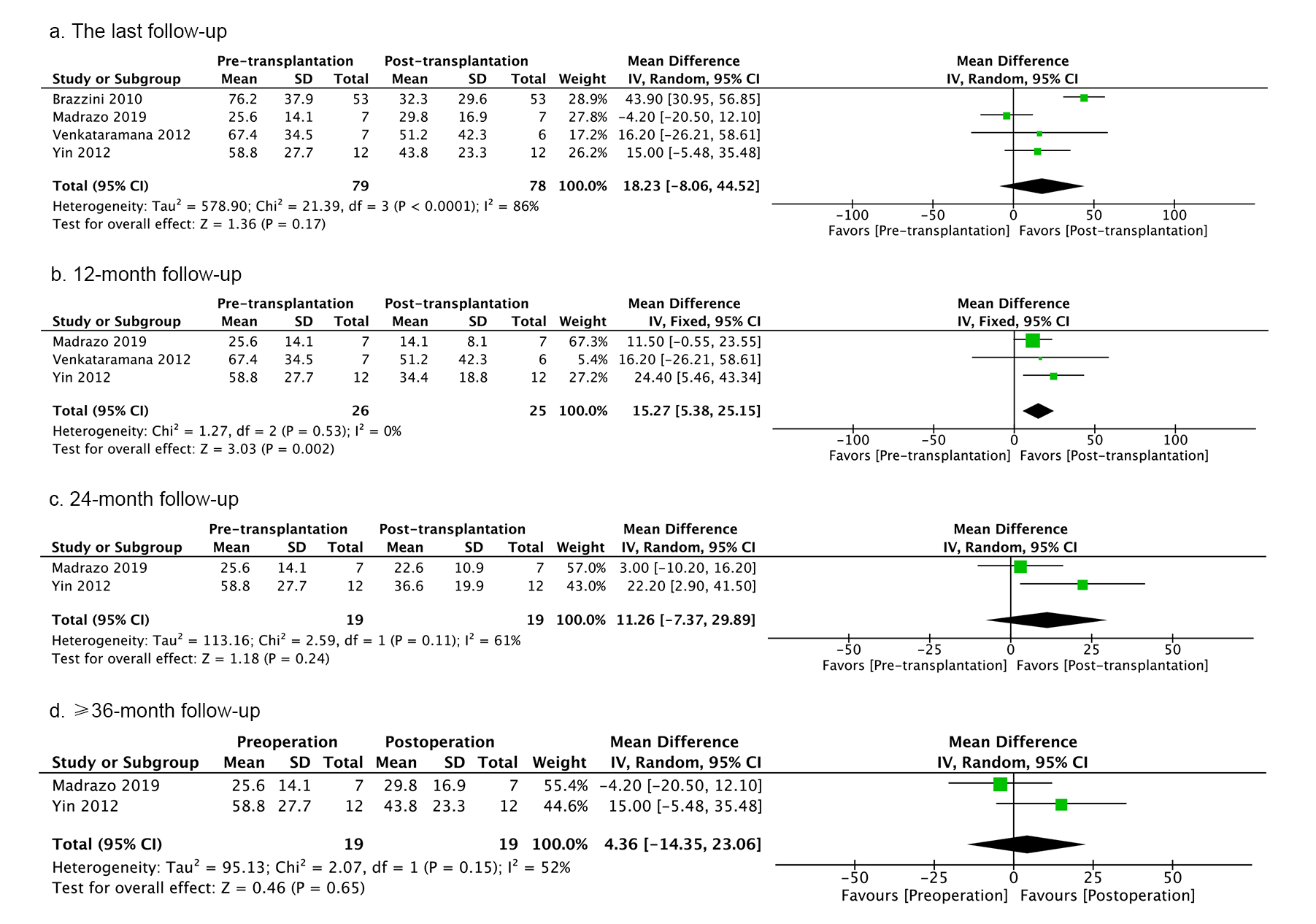

Supplement: Supplementary file 2 — Additional file 2: Fig. S2. UPDRS score pre- versus post-transplantation in the ‘on’ state at the last follow-up, or at 12-, 24-, and ≥ 36-month follow-ups. The number of studies included are 4, 3, 2, and 2, respectively. If the I2 value is less than 30%, a fixed-effect model is used. If the I2 value is greater than 30%, a random-effect model is used. The sizes of squares represent the weight that each study contributes. The diamond at the bottom represents the overall effect. CI = confidence interval (represented by the lines). [file 12967_2023_4484_MOESM2_ESM.tif]

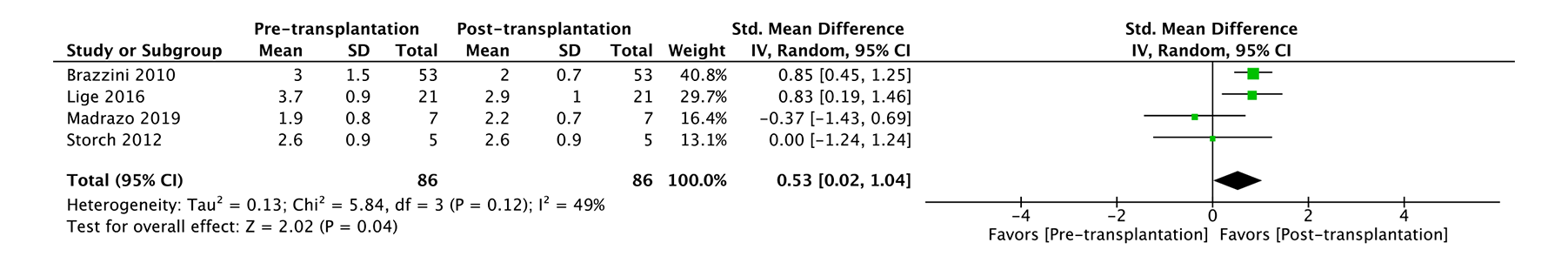

Supplement: Supplementary file 3 — Additional file 3: Fig. S3. H-Y score pre- versus post-transplantation in the ‘on’ or ‘off’ states at the last follow-up. Four studies are included. Random-effect model is used. The sizes of squares represent the weight that each study contributes. The diamond at the bottom represents the overall effect. CI = confidence interval (represented by the lines). [file 12967_2023_4484_MOESM3_ESM.tif]

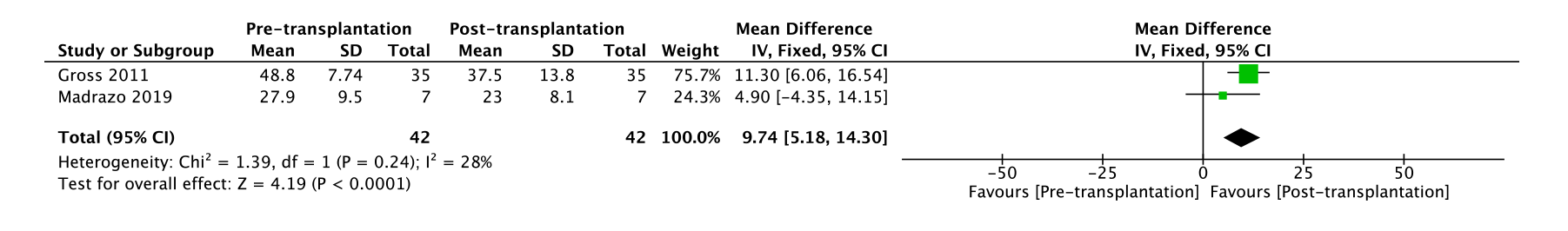

Supplement: Supplementary file 4 — Additional file 4: Fig. S4. UPDRSIII score pre- versus post-transplantation in the ‘off’ state at 48-month follow-up. Two studies are included. Fixed-effect model is used. The sizes of squares represent the weight that each study contributes. The diamond at the bottom represents the overall effect. CI = confidence interval (represented by the lines). [file 12967_2023_4484_MOESM4_ESM.tif]

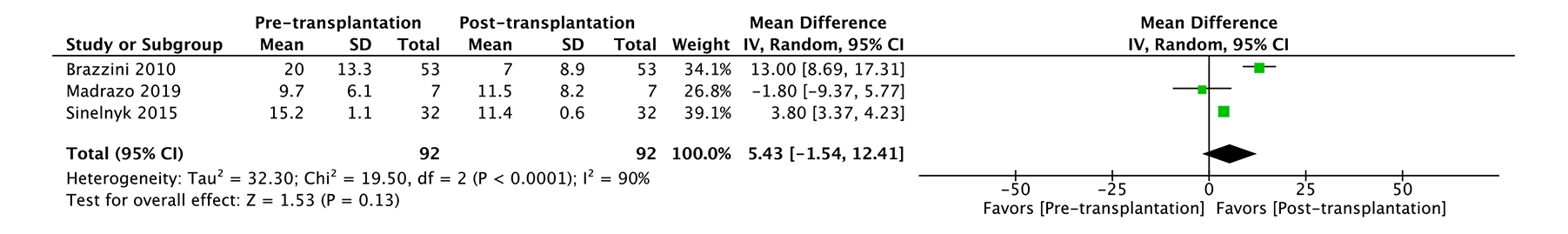

Supplement: Supplementary file 5 — Additional file 5: Fig. S5. Beck Depression inventory score pre- versus post-transplantation in the ‘on’ or ‘off’ states at the last follow-up. Three studies are included. Random-effect model is used. The sizes of squares represent the weight that each study contributes. The diamond at the bottom represents the overall effect. CI = confidence interval (represented by the lines). [file 12967_2023_4484_MOESM5_ESM.tif]

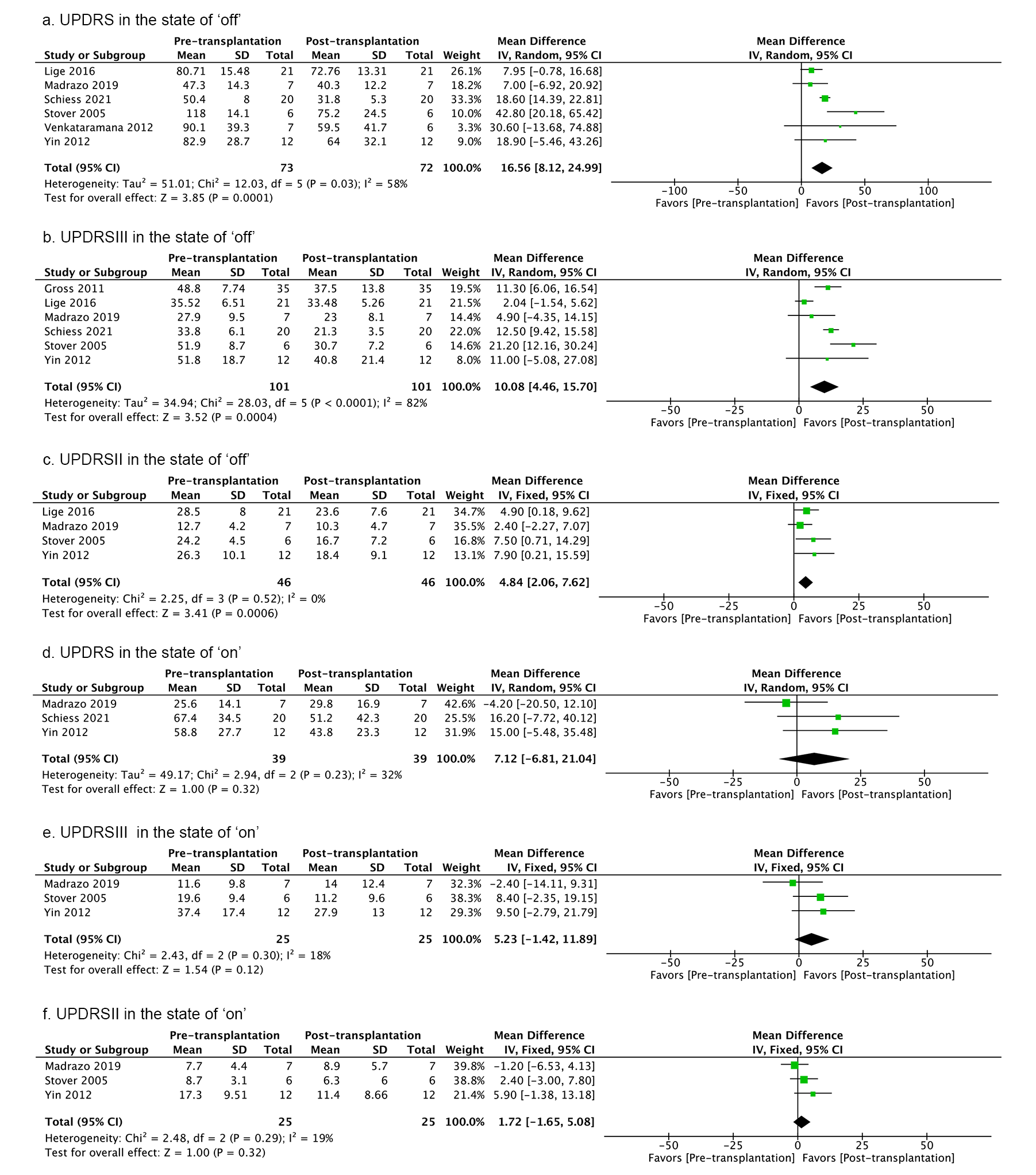

Supplement: Supplementary file 6 — Additional file 6: Fig. S6. UPDRS, UPDRSIII and UPDRSII scores pre- versus post-transplantation in the ‘off’ and ‘on’ states at the last follow-ups after allogeneic cell treatment. The number of studies included are 6, 6, 4, 3, 3, and 3, respectively. If the I2 value is less than 30%, a fixed-effect model is used. If the I2 value is greater than 30%, a random-effect model is used. The sizes of squares represent the weight that each study contributes. The diamond at the bottom represents the overall effect. CI = confidence interval (represented by the lines). [file 12967_2023_4484_MOESM6_ESM.tif]

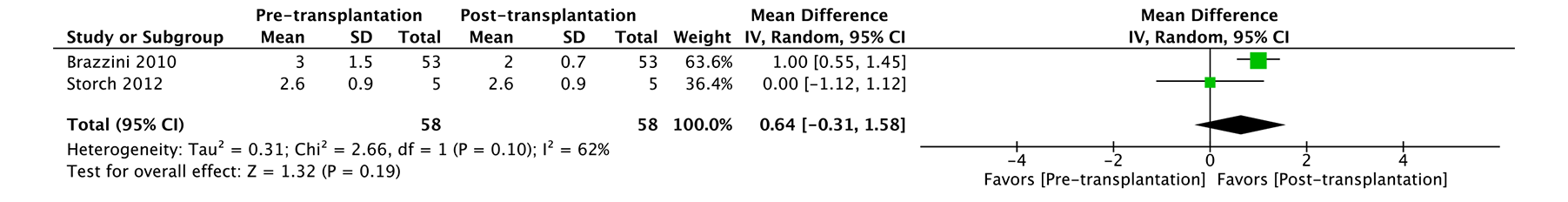

Supplement: Supplementary file 7 — Additional file 7: Fig. S7. H-Y score pre- versus post-transplantation in the ‘on’ or ‘off’ states at the last follow-up after autologous cell treatment. Two studies are included. Random-effect model is used. The sizes of squares represent the weight that each study contributes. The diamond at the bottom represents the overall effect. CI = confidence interval (represented by the lines). [file 12967_2023_4484_MOESM7_ESM.tif]

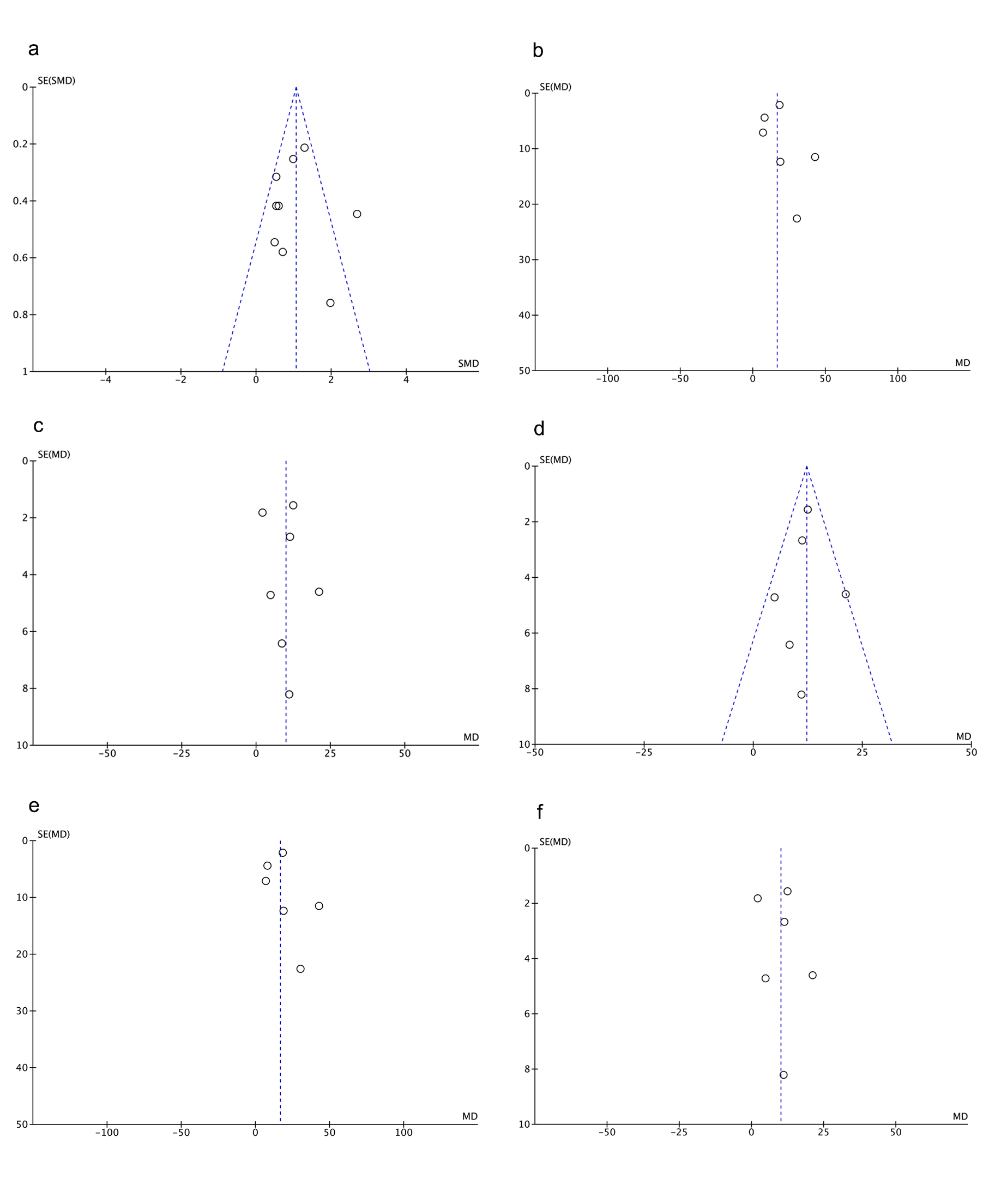

Supplement: Supplementary file 8 — Additional file 8: Fig. S8. Funnel plots assessing potential publication bias on homogeneous cell transplantation in PD treatment. (a) UPDRS or UPDRSIII scores pre- versus post-transplantation in ‘on’ or ‘off’ state at the last follow-up. (b) UPDRS score pre- versus post-transplantation in the ‘off’ state at last follow-up. (c) UPDRSIII score pre- versus post-transplantation in the ‘off’ state at last follow-up. (d) UPDRSIII scores pre- versus post-transplantation in the ‘off’ states at the last follow-up with levodopa responders. (e) UPDRS scores pre- versus post-transplantation in the ‘off’ states after allogeneic cell treatment. (f) UPDRSIII scores pre- versus post-transplantation in the ‘off’ states at the last follow-ups after allogeneic cell treatment. Each dot represents a single study. The dashed vertical line represents the pooled effect size. The dashed diagonal lines represent 95% confidence limits around the pooled effect size for each standard error on the vertical axis, and are only provided in plots when fixed effect models were used. [file 12967_2023_4484_MOESM8_ESM.tif]
