# Supplementary material for: Cell-therapy for Parkinson’s disease: a systematic review and meta-analysis
Source: J Transl Med. 2023 Sep 7;21:601. doi: 10.1186/s12967-023-04484-x (PMC10483810; doi:10.1186/s12967-023-04484-x)
Supplement: Supplementary file 9 — Additional file 9: Table S1. Fetal mesencephalic tissue transplantation: characteristics of the studies and subjects. [file 12967_2023_4484_MOESM9_ESM.docx]

Table S1 Fetal mesencephalic tissue transplantation: characteristics of the studies and subjects

| Study | Patients in transplant group | | | Graft Location | Follow-up  (months) | Outcome | Study Type | Origin |
| --- | --- | --- | --- | --- | --- | --- | --- | --- |
|  | N | Age (years) | Disease Duration (year) |  |  |  |  |  |
| McRae 2018^a 13^ | 19 | 56.9±10.3 | 13.4±5.0 | bilateral putamen | 4, 8, 12 | (11) | RCT, multi-center | USA |
| Ma 2010^a 14^ | 33 | 57.2±9.9 | 13.8±5.3 | bilateral putamen | 12, 24, 48 | (3), (15) | RCT, multi-center | USA |
| McRae 2004^a 15^ | 12 | 59.9±7.9 | 15.5±6.6 | bilateral putamen | 4, 8, 12 | (1), emotional and social functioning | RCT, multi-center | USA |
| Gordon 2004^a 16^ | 19 | 56.9±10.3 | 13.4±5.0 | bilateral putamen | 4, 12 | reaction time and movement time | RCT, multi-center | USA |
| Trott 2003^a 17^ | 19 | 56.9±10.3 | 13.4±5.0 | bilateral putamen | 12 | (2), (8) | RCT, multi-center | USA |
| McRae 2003^a 18^ | 12 | 59.9±7.9 | 15.5±6.6 | bilateral putamen | 12, 24 | NEO Five-Factor Inventory (NEO-FFI) | RCT, multi-center | USA |
| Ma 2002^a 19^ | 17 | NP | NP | bilateral putamen | 12, 24 | (1), (15) | RCT, multi-center | USA |
| Freed 2001^a 20^ | 19 | 56.9±10.3 | 13.4±5.0 | bilateral putamen | 4, 8, 12 | (1), (5), (15) | RCT, multi-center | USA |
| Nakamura 2001^a 21^ | 19 | 56.9±10.3 | 13.4±5.0 | bilateral putamen | 12 | (3), (15) | RCT, multi-center | USA |
| Olanow 2009^b 22^ | 23 | 58.5±8.4 | NP | bilateral putamen | 3, 6, 12, 18, 24 | dyskinesia score | RCT, multi-center | USA |
| Olanow 2003^b 23^ | 23 | 58.5±8.4 | NP | bilateral putamen | 1, 3, 6, 9, 12, 15, 18, 21, 24 | (1) | RCT, multi-center | USA |
| Pogarell 2006^c 24^ | 2 | 47.0±8.5 | 14.0±1.4 | bilateral caudate nucleus and putamen | 36, 96 | (2), (3), (18) | prospective, single-center, case study | Germany |
| Brundin 2000^c 25^ | 5 | 53.0±9.8 | 12.6±1.5 | bilateral caudate nucleus and putamen | 10-23 | (3), (10), (15) | prospective, multi-center, uncontrolled clinical trial | Sweden, England, Germany |
| Hagell 2000^c 26^ | 5 | 53.0±9.8 | 12.6±1.5 | bilateral caudate nucleus and putamen | 3, 7, 12, 24 | Nottingham Health Profile (NHP) | prospective, multi-center, uncontrolled clinical trial | Sweden, England, Germany |
| Piccini 2000^c 27^ | 4 | 49.2±5.9 | 12.8±1.7 | bilateral caudate nucleus and putamen | 18 | (1), (3), (15) | prospective, multi-center, uncontrolled clinical trial | Sweden, England |
| Hagell 1999^c 28^ | 5 | 48.8±4.6 | 11.6±3.3 | bilateral caudate nucleus and putamen | 1-6, 7-12, 13-24 | (1), (3), (15) | prospective, multi-center, uncontrolled clinical trial | Sweden, England |
| Wenning 1997^c 29^ | 6 | 50.5±5.1 | 9.7±2.6 | unilateral putamen or putamen and caudate | 10-72 | (3), (10), (15) | prospective, multi-center, uncontrolled clinical trial | Sweden, England |
| Lindvall 1994^c 30^ | 2 | 48, 58 | 12, 9 | unilateral putamen | 1-36 | (6), (10), (15) | prospective, single-center, case study | Sweden |
| Sawle 1992^c 31^ | 2 | 48, 58 | 12, 9 | unilateral putamen | 5, 8, 13  7, 12 | (15) | prospective, single-center, case study | Sweden |
| Lindvall 1992^c 32^ | 2 | 48, 58 | 12, 9 | unilateral putamen | 1, 2, 3, 4, 5, 6, 7, 8, 9, 10, 11, 12 | (6), (10) | prospective, single-center, case study | Sweden |
| Lindvall 1989^c 33^ | 2 | 48, 55 | 14, 14 | unilateral caudate nucleus and putamen | 1, 2, 3, 4, 5, 6 Weeks | (6), (10), (15), neuropsychological examination | prospective, single-center, case study | Sweden |
| Lindvall 1988^c 34^ | 2 | 48, 55 | NP | unilateral caudate nucleus and putamen | 3, 6 | (10), (15) | prospective, single-center, case study | Sweden |
| Schumacher 2000^d 35^ | 12 | 60.8±6.5 | 14.0±5.9 | unilateral striatal | 3, 6, 9, 12 | (1), (2), (3), (4), (14), (15) | prospective, single-center, uncontrolled clinical trial | USA |
| Fink 2000^d 36^ | 12 | 60.8±6.5 | 14.0±5.9 | unilateral striatal | 12 | (1), (3), (14), (15) | prospective, single-center, uncontrolled clinical trial | USA |
| Deacon 1997^d 37^ | 1 | 69 | 15 | right striatal | 7 | immunohistochemical studies at autopsy | retrospective, single-center, case study | USA |
| Jacques 1999^e 38^ | 60 | 57±8 | 12±5 | bilateral putamen | 12 | (1) | prospective, single-center, uncontrolled clinical trial | USA |
| Kopyov 1997^e 39^ | 13 | 56.4±8.0 | 11.7±3.6 | bilateral putamen | 6 | (1), (4), (10) | prospective, single-center, controlled clinical trial | USA |
| Kopyov 1996^e 40^ | 22 | 55.2±8.2 | 12.0±4.1 | unilateral or bilateral caudate nucleus and putamen | 6-24 | (1), (4), (7), (8), (10), (14) | prospective, single-center, uncontrolled clinical trial | USA |
| Defer 1996^f 41^ | 5 | 57.4±7.9 | 17.2±3.3 | unilateral putamen or putamen and caudate | 15-36 | (6), (15) | prospective, NP, uncontrolled clinical trial | France |
| Remy 1995^f 42^ | 5 | 57.4±7.9 | 12.0±4.1 | unilateral putamen or putamen and caudate | 3, 6, 12, 18, 24 | (14), (15) | prospective, NP, uncontrolled clinical trial | France |
| Peschanski 1994^f 43^ | 2 | 56.0±9.9 | 13.5±4.9 | unilateral putamen or putamen and caudate | 3, 6-7, 12 | (6), (10), (15) | prospective, NP, case study | France |
| Sass 1995^g 44^ | 4 | 53.5±10.4 | 15.0±4.1 | unilateral or bilateral caudate nucleus | 12, 24, 26 | (8) | prospective, single-center, uncontrolled clinical trial | USA |
| Price 1995^g 45^ | 9 | 54.7±19.3 | 13.2±4.5 | unilateral or bilateral caudate | 1, 3, 6, 12 | (7) | prospective, single-center, uncontrolled clinical trial | USA |
| Spencer 1992^g 46^ | 4 | 52.0±7.4 | 14.3±4.8 | unilateral caudate nucleus | 3-18 | (1), (2), (3), (4), (5), (7), (14), (15), cerebrospinal fluid homovanillic acid | RCT, single-center | USA |
| Henderson 1992^h 47^ | 7 | 57.4±5.2 | 17.9±4.3 | right caudate nucleus | 6-9 | visual evoked cortical responses and electroretinograms | prospective, NP, uncontrolled clinical trial | UK |
| Henderson1991^h 48^ | 12 | 56.0±6.3 | 17.2±6.1 | right caudate nucleus | 3, 6, 12 | (6), (12), (13) | prospective, NP, uncontrolled clinical trial | UK |
| Hitchcock 1990^h 49^ | 12 | 56.0±6.3 | 17.2±6.1 | right caudate nucleus | 1, 3, 6, 9, 12 | (10), (12), (13) | prospective, NP, uncontrolled clinical trial | UK |
| Hitchcock 1988^h 50^ | 2 | 60, 45 | 25, 10 | right caudate nucleus | 1-3 | (4), (12), (13) | prospective, single-center, case study | UK |
| Hauser1999^i 51^ | 6 | 55.9±9.3 | 18.2±7.6 | bilateral putamen | 1, 3, 6, 9, 12, 18, 24 | (1), (5), (6), (10), (14), (15) | prospective, single-center, uncontrolled clinical trial | USA |
| Freeman 1995^i 52^ | 4 | 52.3±10.0 | 14.3±5.9 | bilateral putamen | 1, 3, 6 | (1), (4), (5), (10), (14), (15) | prospective, single-center, uncontrolled clinical trial | USA |
| Kordower 1996^i 53^ | 1 | 59 | 8 | bilateral putamen | 18 | histologic studies at autopsy | retrospective, single-center, case study | USA |
| Kordower 1995^i 54^ | 1 | 59 | 8 | bilateral putamen | 1, 3, 6, 9, 12,15 | (1), (15), immunohistochemical studies at autopsy | retrospective, single-center, case study | USA |
| Barker 2019 ^55^ | 11 | NP | NP | bilateral putamen | 36 | (3), (6), (9), (10), (15) | RCT, multi-center | UK |
| Li 2010 ^56^ | 1 | 49 | NP | bilateral putamen | 16 years | pathological studies at autopsy | retrospective, single-center, case study | Sweden |
| Kordower 2008 ^57^ | 1 | 63 | 25 | bilateral putamen | 14 years | immunohistochemical studies at autopsy | retrospective, single-center, case study | USA |
| Kordower 2008 ^58^ | 1 | 61 | 22 | bilateral putamen | 14 years | pathological studies at autopsy | retrospective, single-center, case study | USA |
| Li 2008 ^59^ | 2 | 48, 43 | 12, 5 | bilateral putamen, bilateral putamen and caudate | 13 years,  16 years | immunohistochemical and pathological studies at autopsy | retrospective, multi-center, case study | Sweden, England |
| Mendez 2008 ^60^ | 5 | NP | NP | NP | 9-14 years | pathological studies at autopsy | retrospective, NP, case study | NP |
| Cochen 2003 ^61^ | 6 | 55.3±5.9 | 13.5±7.3 | bilateral putamen | 24 hours | (3), (15), (19) | prospective, NP, uncontrolled clinical trial | France |
| Wu 2002 ^62^ | 5 | 53-64 | NP | right caudate nucleus | 3, 9, 14 | (12) | retrospective, single-center, case study | China |
| Mendez 2000 ^63^ | 2 | 63.5±10.6 | 14.5±3.5 | bilateral putamen | 6, 12, 15 | (1), (4), (5), (10), (15) | prospective, single-center, uncontrolled clinical trial | Canada |
| Ross 1999 ^64^ | 24 | 58±11 | NP | bilateral putamen | 12 | magnetic resonance spectroscopy | RCT, NP | USA |
| Piccini 1999 ^65^ | 1 | 69 | 9 | right putamen | 1, 2, 3, 4, 5, 6, 7, 8, 9, 10 years | (1), (10), (15), (17) | prospective, single-center, case study | UK |
| Kordower 1998 ^66^ | 1 | 61 | 27 | bilateral putamen | 6, 12 | (1), (2), (3), (10), (15), immunohistochemical studies at autopsy | prospective, single-center, case study | USA |
| Mamelak 1998 ^67^ | 1 | 52 | 10 | bilateral putamen | 23 | pathological findings at autopsy, chemistry and pathological studies of cyst fluid | retrospective, single-center, case study | USA |
| Levivier 1997 ^68^ | 3 | NP | NP | unilateral putamen | 6, 9, 12 | (1), (4), (14), (15) | prospective, single-center, case study | France |
| López-Lozano 1997 ^69^ | 10 | 61.0±6.8 | 13.5±3.8 | unilateral caudate nucleus | 1, 6, 12, 36, 60 | (1), (4), (13), (14) | prospective, single-center, uncontrolled clinical trial | Spain |
| Baker 1997 ^70^ | 5 | 58.8±10.1 | 11.8±3.7 | bilateral putamen | 3, 6, 12 | acoustic, electroglottographic, and perceptual measures | prospective, single-center, uncontrolled clinical trial | USA |
| Folkerth 1996 ^71^ | 1 | 52 | 4 | right caudate and left putamen | 23 | histologic studies at autopsy | retrospective, single-center, case study | USA |
| Ząbek 1994 ^72^ | 3 | 50.3±2.5 | 11.7±2.9 | unilateral caudate nucleus | 1, 3, 6, 9, 18, 24, 27, 30 | (6), (10) | prospective, single-center, case study | Poland |
| Markham 1994 ^73^ | 6 | 50.5±7.0 | 13.2±4.7 | unilateral putamen and caudate | 10-15 | (1), (4), (10), (15) | prospective, single-center, case study | USA |
| Molina 1993 ^74^ | 5 | 51 | NP | right putamen and caudate | 3 | (1), (10) | prospective, single-center, uncontrolled clinical trial | Cuba |
| Freed 1992 ^75^ | 7 | 56.3±9.3 | 13.4±5.6 | bilateral putamen or unilateral putamen and caudate | 6, 12 | (3), (5), (15) | prospective, single-center, uncontrolled clinical trial | USA |
| Lindvall 1990 ^76^ | 49 | 49 | 12 | unilateral putamen | 1, 2, 3, 4, 5 | (6), (10), (15) | prospective, single-center, case study | Sweden |
| Freed 1990 ^77^ | 1 | 52 | 20 | right putamen and caudate | 12 | (3), (6), (9), (15) | prospective, single-center, case study | USA |
| Madrazo 1988 ^78^ | 1 | 50 | 9 | right caudate nucleus | 2 | (1) | prospective, single-center, case study | Mexico |

^a, b, c, d, e, f, g, h, i^: label publications each representing the same study; NP = not provided; RCT = randomized controlled trial; n = the number of implant patients; CG = control group (1) = Unified PD Rating Scale (UPDRS); (2) = UPDRSII; (3) = UPDRSIII; (4) = Hoehn and Yahr (H&Y) staging; (5) = Schwab and England scale; (6) = time tests for specified actions; (7) = Beck Depression Inventory or Beck Anxiety Inventory or other psychiatric scale; (8) = Mini-mental State Examination (MMSE) or other cognitive scale; (9) = PDQ-39 or Activities of Daily Living Scale; (10) = the time in “off” or “on” state; (11) Global Rating Scale; (12) Webster Rating Scale; (13) = Northwestern University Disability Scale (NUDS); (14) = Magnetic Resonance (MR) Imaging; (15) = PET Molecular Imaging ([^18^F]-FDOPA); (16) = PET Molecular Imaging (vesicular monoamine transporter 2, VMAT2); (17) = PET Molecular Imaging (^11^C-β-CFT); (18) = N-(3-iodopropen-2-yl)-2à-carbomethoxy-3à-(4-chloro- phenyl) tropane (IPT) SPECT ([^123^I]-IPT SPECT); (19) = fluorethyl-methyl-2β- carboxymethoxy-3β-4-bromophenyl-tropane ([^76^Br]-FE-CBT PET)
