# Supplementary material for: Cell-therapy for Parkinson’s disease: a systematic review and meta-analysis
Source: J Transl Med. 2023 Sep 7;21:601. doi: 10.1186/s12967-023-04484-x (PMC10483810; doi:10.1186/s12967-023-04484-x)
Supplement: Supplementary file 10 — Additional file 10: Table S2. Adrenal medulla transplantation: characteristics of the studies and subjects. [file 12967_2023_4484_MOESM10_ESM.docx]

Table S2 Adrenal medulla transplantation: characteristics of the studies and subjects

| Study | Patient in transplant group | | | Graft Location | Follow-up  (months) | Outcome | Study Type | Origin |
| --- | --- | --- | --- | --- | --- | --- | --- | --- |
|  | N | Age (years) | Disease Duration (year) |  |  |  |  |  |
| Porena 1996^a 79^ | 13 | NP | NP | right caudate nucleus | 3, 6, 12, 24 | (5), (11), urological profiles | prospective, single-center, case study | Italy |
| Sydow 1995^a 80^ | 2 | 49, 62 | 14, 11 | unilateral putamen | 12, 24 | (1), (2), (3), (4), (5), (6) | prospective, single-center, case study | Sweden |
| Diamond 1994^b 81^ | 4 | 49.5±5.3 | 11.8±3.8 | right caudate nucleus | 2-48 | (1), (4), (10) | prospective, single-center, case study | USA |
| Goetz 1991^b 82^ | 61 | 54.0±9.0 | 14.6±6.4 | unilateral or bilateral caudate, or unilateral thalamus | 12, 24 | (1), (2), (3), (4), (5), (10) | prospective, multi-center, uncontrolled clinical trial | USA, Canada |
| Jimenez 1994^c 83^ | 8 | NP | NP | right caudate nucleus | 1, 6, 12 | (1), (4), (5) | RCT, single-center | Mexico |
| Madrazo 1991^c 84^ | 5 | 45.0±10.9 | 6.6±2.3 | right caudate nucleus | 12, 36 | (1), neuropsychological scale, electrophysiological data | prospective, single-center, case study | Mexico |
| Velasco 1991^c 85^ | 10 | 48.4 | 5.8 | right caudate nucleus | 12 | (1), (4) | prospective, single-center, uncontrolled clinical trial | Mexico |
| Olson 1991 ^86^ | 1 | 63 | 19 | left putamen | 2-3, 7, 13 | (6), evoked potentials | prospective, single-center, case study | Sweden |
| Kordower 1991 ^87^ | 1 | 48 | 15 | right caudate nucleus | 2, 6, 12, 14, 18, 24, 30 | (4), histological and neuropathological features at autopsy | prospective, single-center, case study | USA |
| López-Lozano 1991 ^88^ | 20 | 58±NP | 13.5±NP | right caudate nucleus | 2, 5, 7 | (1), (4), (5), (13) | prospective, single-center, uncontrolled clinical trial | Spain |
| Skinner 1990 ^89^ | 10 | 53±NP | 10.5±NP | caudate | NP | NP | prospective, single-center, uncontrolled clinical trial | USA |
| Goetz 1989 ^90^ | 19 | 53.8±8.0 | 12.9±7.4 | right caudate nucleus | 1, 3, 6 | (1), (2), (3), (4), (5), (10) | prospective, multi-center, uncontrolled clinical trial | USA |
| Penn 1988 ^91^ | 5 | 49.2±NP | 10.6±NP | right caudate nucleus | 1, 2, 3, 4, 5 | (3), (4), (5), (10) | prospective, single-center, uncontrolled clinical trial | USA |
| Lindvall 1987 ^92^ | 2 | 46, 63 | 14, 10 | right putamen | 2 | (6), (7), (11), evoked potentials, cerebral blood flow recordings, self-scoring grades, monoamine metabolites in the CSF | prospective, single-center, case study | Sweden |
| Madrazo 1987 ^93^ | 2 | 37, 39 | NP | right caudate nucleus | 1, 2, 3, 4, 5, 6, 7, 8, 9, 10 | electromyographic | retrospective, single-center, case study | Mexico |
| Backlund 1985 ^94^ | 2 | 55, 46 | 8, 5 | right caudate nucleus | 12-24 | self-scoring grades, monoamine metabolites in the CSF | prospective, single-center, case study | Sweden |

^a, b, c^: label publications each representing the same study ; NP = not provided; RCT = randomized controlled trial; n = the number of implant patients; CG = control group (1) = Unified PD Rating Scale (UPDRS); (2) = UPDRSII; (3) = UPDRSIII; (4) = Hoehn and Yahr (H&Y) staging; (5) = Schwab and England scale; (6) = time tests for specified actions; (7) = Beck Depression Inventory or Beck Anxiety Inventory or other psychiatric scale; (8) = Mini-mental State Examination (MMSE) or other cognitive scale; (9) = PDQ-39; (10) = the time in “off” or “on” state; (11) Global Rating Scale; (12) Webster Rating Scale; (13) = Northwestern University Disability Scale (NUDS); (14)= Magnetic Resonance (MR) Imaging; (15) = PET Molecular Imaging ([^18^F]-FDOPA);
